# Antenatal ultrasound needs-analysis survey of Australian rural/remote healthcare clinicians: recommendations for improved service quality and access

**DOI:** 10.1186/s12889-023-17106-4

**Published:** 2023-11-17

**Authors:** Amber Bidner, Eva Bezak, Nayana Parange

**Affiliations:** 1 Allied Health and Human Performance, The University of South Australia, Corner of North Terrace and Frome Road, Adelaide, SA 5001 Australia; 2 Department of Physics, The University of Adelaide, North Terrace, Adelaide, SA 5001 Australia

**Keywords:** Antenatal, Obstetrics, Ultrasound (US), Point-of-Care Ultrasound (PoCUS), Medical education/training, Service equality, Rural/remote, Low-resource setting

## Abstract

**Background:**

Ultrasound is the primary diagnostic tool in pregnancy, capable of identifying high-risk pregnancies and life-threatening conditions, allowing for appropriate management to prevent maternal and fetal morbidity and mortality. Women and babies from rural and remote Australia and low-resource areas worldwide experience poorer health outcomes and barriers to accessing antenatal care and imaging services. Healthcare clinicians working in these regions face significant challenges practising with limited resources and accessing training opportunities.

**Objective:**

To perform an exploratory needs-analysis survey investigating the availability, accessibility and use of antenatal ultrasound in rural Australia, exploring rural clinicians’ interest in and access to ultrasound training opportunities.

**Methods:**

The survey tool for this cross-sectional study was designed and distributed as an anonymous online questionnaire targeting healthcare clinicians (doctors, nurses, midwives, clinic managers, Aboriginal healthcare workers) providing antenatal care in rural regions. Descriptive analysis was applied to quantitative data and thematic analysis was used to explore qualitative components.

**Results:**

A total of 114 valid survey responses were analysed. Overall, 39% (43/111) reported ultrasound was not used when providing antenatal care to patients at their clinic, stating ‘Lack of ultrasound equipment (73%,29/40) and inaccessibility of training opportunities (47%,19/40) as the main reasons. For those with ultrasound (61%,68/111), estimating due date (89%,57/64) was the main use, and limited training/skills to operate the equipment (59%,38/64) and inaccessibility/distance of training opportunities (45%,29/64) were the most commonly reported barriers. Clinicians described a lack of childcare options (73%,74/102), long distances to reach ultrasound services (64%,65/102), appointment (59%,60/102) and transport availability/times (46%,47/102) as the main obstacles to patient access. Increased attendance, compliance with care directives, parental bonding and improved lifestyle choices were described by respondents as positive outcomes of antenatal ultrasound use.

**Conclusions:**

Future efforts to combat inequitable service access must adopt a coordinated approach to meet the needs of pregnant women in low-resource settings. Providing portable ultrasound equipment, training in antenatal Point-of-Care ultrasound (PoCUS) with ongoing support/mentoring and accreditation of health professionals could strengthen rural workforce capacity. This, along with addressing the complex economic, environmental and socio-cultural barriers faced by patients, could improve service access and pregnancy outcomes in rural and remote communities.

**Supplementary Information:**

The online version contains supplementary material available at 10.1186/s12889-023-17106-4.

## Introduction

Maternal and infant mortality rates, low birthweight and preterm births are substantially higher in rural Australia compared to metropolitan areas, and rates of neonatal death increase with increasing remoteness [1–3]. These differences are even more significant for Aboriginal populations who continue to experience higher perinatal and maternal mortality and morbidity compared to non-indigenous populations [3] (see Supplementary Tables S1 and S2 for perinatal, stillbirth and neonatal mortality rates and maternal mortality ratio by Indigenous status and remoteness area [4, 5]). The key risk factors associated with infant mortality and morbidity include low birthweight, maternal health and behaviours (smoking, alcohol, nutrition during pregnancy), socio-economic status, indigenous status and inaccessibility of healthcare services (geographical isolation), including antenatal ultrasound [1, 4, 6]. The Australian Department of Health and Aged Care and the Royal Australian and New Zealand College of Obstetricians and Gynaecologists (RACGP) recommends all women be offered an obstetric ultrasound performed by a qualified sonographer or sonologist before 20 weeks gestation to confirm or determine gestational age, detect multiple pregnancies and screen for congenital conditions [1, 7] (see Supplementary Table S3 for formal antenatal ultrasound schedule and screening components).

In rural Australia, fewer pregnant women have access to antenatal care and ultrasound services compared to women in metropolitan areas. In a 2018 Senate inquiry into the ‘*availability and accessibility of diagnostic imaging in Australia*’, limited access and additional expenses incurred by patients using ultrasound services in “*regional, rural and remote areas*” were highlighted and concerns raised over the quality and age of available imaging equipment [8]. Rural Australia has suffered from a shortage of trained sonographers for over a decade. Many remote medical centres have no onsite or local sonographer, relying on fly-in fly-out professionals attending as infrequently as one day per month resulting in significant delays in patient access, diagnosis and treatment. This can be critical in the remote setting where considerable time and logistical planning may be needed to reach obstetric care services. The past two decades have seen the closure of many maternity services and these closures correlate with rurality and an increase in unplanned out-of-hospital births and adverse outcomes [9]. Radiologists needed to report ultrasound images are also in short supply [8]. Telehealth technologies may help in this regard to allow for off-site image interpretation/reporting and can also be useful for training and supervision/guidance of remote clinicians [8, 10, 11].

One recommendation from the Senate’s 2018 report [8] was to expand the clinical scope of practice for nurses to include certain ultrasound services with appropriate training where qualified sonographers are unavailable. In Australia, diagnostic ultrasound is primarily performed by sonographers and reported by radiologists. Sonographers require years of postgraduate training and supervised practice to become qualified and registered to practice with the Australian Sonographer Accreditation Registry [12]. In contrast, Point-of-care ultrasound (PoCUS) serves to answer a specific clinical question and is performed by healthcare workers who are not medical imaging professionals [12]. Despite ultrasound being a highly-skilled, operator-dependent modality [13], PoCUS may be performed in Australia and throughout most of the world by healthcare workers with limited/no training or formal accreditation/registration [14, 15]. This, along with the challenges to delivering equitable health care in rural locations has made PoCUS an attractive option in resource-poor settings [16]. However, PoCUS performed by untrained operators can present a potential risk to patients and subsequent financial burden to healthcare systems in cases of misdiagnoses. An inadequate examination could mean false-negative results, while false-positive results could lead to unnecessary examinations, specialist referrals, costly patient transfers and considerable patient anxiety [14]. Point-of-Care ultrasound can assist with the skills and service deficit in rural and remote areas [17]. However, research has shown that even where ultrasound equipment is available, clinicians often lack the training and supervision/mentorship to effectively use it [18]. For Australian rural clinicians who choose to upskill, additional barriers are faced accessing existing PoCUS training courses (mostly city-based) and fulfilling accreditation requirements (‘Certificate in Allied Health Performed Ultrasound’ [19], or a ‘Certificate in Clinician Performed Ultrasound’ [20] for doctors, administered by the Australasian Society for Ultrasound in Medicine/ASUM) that require some assessments be performed under the direct supervision of an expert [19, 20]. Additionally, rural women face their own challenges. Many have difficulties accessing antenatal care due to distance, transport and service availability, and may be required to deliver away from their community. This entails additional costs, limited family/emotional support, separation from other children, a lack of service continuity and potentially inappropriate or culturally unsafe care [9, 21].

This descriptive exploratory research aimed to administer a cross-sectional needs analysis survey to investigate the availability, accessibility and use of antenatal ultrasound in rural Australia. The perceptions and opinions of rural clinicians regarding patient access to ultrasound services and their interest in and access to ultrasound training opportunities were explored. Recommendations and strategies to combat inequitable service access, informed by frontline rural healthcare workers are provided.

## Methods

### Survey development

The needs analysis survey tool was developed for this cross sectional study in 2019 by a multidisciplinary team from UniSA to investigate the availability, accessibility and use of antenatal ultrasound in rural Australia, and explore the perceptions of rural clinicians regarding access to and interest in PoCUS training opportunities. The questions were informed by the research team's clinical experience and the results of a scoping review [22]. Content validation of the draft survey was conducted by four experienced healthcare professionals (fully qualified healthcare professionals with minimum 2 years clinical practice, currently working in antenatal care and in rural practice within 2 years) in their respective fields of sonography, nursing and midwifery, rural/remote health, and Aboriginal health. They assessed the relevance and clarity of the survey questions and usability of the tool. An internal pilot was conducted among a sample of regional, rural and remote healthcare professionals who provided feedback on the survey questions and practicality. The survey was then distributed using a census-based sampling method within rural South Australia (SA) to targeted facilities [23]. The internal pilot and SA-based surveys (21 SA healthcare professionals) were content analysed with responses reviewed for consistency and reliability. The survey tool for national distribution was modified based on these responses, and internally validated by a team of researchers at UniSA and representatives of the target demographic. Cultural appropriateness was assessed. The final survey was released as an anonymous online questionnaire using Survey Monkey, and included a total of 46 questions (multiple choice, multiple-response, free/open-text response, and Likert-scale formats). Participants were not required to answer all questions; logic pathways depended on the clinic's use of ultrasound, availability of ultrasound equipment and the respondents’ interest in training (see Supplementary material needs-analysis survey pro forma).

### Participant recruitment and survey distribution

Clinicians in regions zoned RA2 to RA5 under the Australian Statistical Geography Standard (ASGS) Accessibility Remoteness Index of Australia (ARIA) [24, 25] were targeted for the survey, including clinic managers, doctors, nurses, midwives, Aboriginal healthcare workers and community healthcare workers, representative of the primary clinicians planning and providing antenatal care in rural clinics. The ASGS ARIA defines five geographical categories or remoteness areas (RAs) determined by road distance from the closest urban centre (see Supplementary Fig. S1 Map of the ASGS ARIA + 2016 Remoteness Areas [26]). The term ‘rural’ within this manuscript refers to areas outside Major cities (RA1), i.e. RA2 to RA5 of the ASGS ARIA + 2016. The term ‘remote’ encompasses more isolated areas, i.e. RA4 and RA5 regions. A non-probability sampling method with self-selection/voluntary response was used with multiple recruitment methods. Precontact was sought through direct cold-calling of 162 targeted clinics publicly listed on the Australian Indigenous Health Info Net, with up to 5 repeat call attempts. From this, 76 clinics were contacted and 57 personal emails provided by clinic managers (many services operated with a primary clinic managing multiple satellite and outreach clinics). The survey information and link were sent by email to clinic managers who agreed to distribute to their staff (tiered/respondent-driven recruitment). Professional organisations (The RACGP, National Aboriginal Community Controlled Health Organisation, Australian College of Midwives, Council of Remote Area Nurses of Australia) agreed to disseminate the invitation to participate to their members through industry newsletters/e-magazines/journals, websites, communication platforms and members email lists. Open advertising targeting rural clinicians was used through all state, territory, Rural and SIG- Pre and post-natal branches of the RACGP. The survey was open from September 2019 to February 2020 to capture transient rural workers and spanning 2 medical rotation periods. Multiple reminders were sent over this period by personal email. The completely anonymous survey design aimed to encourage participation and candid responses, and a $1,000 random draw incentive to finance clinical training or conference attendance was offered. The survey's relevance to rural healthcare workers was emphasized through initial clinic phone calls and in altruistic text appeal in advertising and the survey invitation email and pre-ample.

### Analysis

Descriptive analysis of quantitative data was performed using Excel 16.0 (Microsoft Corp., Microsoft Excel, 2016, Redmond, Washington, USA) and SPSS 27.0 (IBM Corp., SPSS Statistics for Windows, 2020, Armonk, NY, USA). Response frequencies were grouped and analysed by professional role and location of practice (remoteness area). Data (graphs/figures) was displayed using GraphPad Prism 8.2.0 (GraphPad Software Inc., GraphPad Prism, 2019, La Jolla, CA, USA). The qualitative survey components were analysed using Nvivo (QSR International, Nvivo, 2020, Burlington, Massachusetts, USA) and a thematic analysis used to highlight relevant themes and concepts. Respondent quotations are provided throughout and in the Supplementary material.

Only healthcare professionals were involved in the survey. This research was granted ethics approval by The UniSA’s Human Research Ethics Committee (Project number – 201543).

## Results

**Fig. 1:**
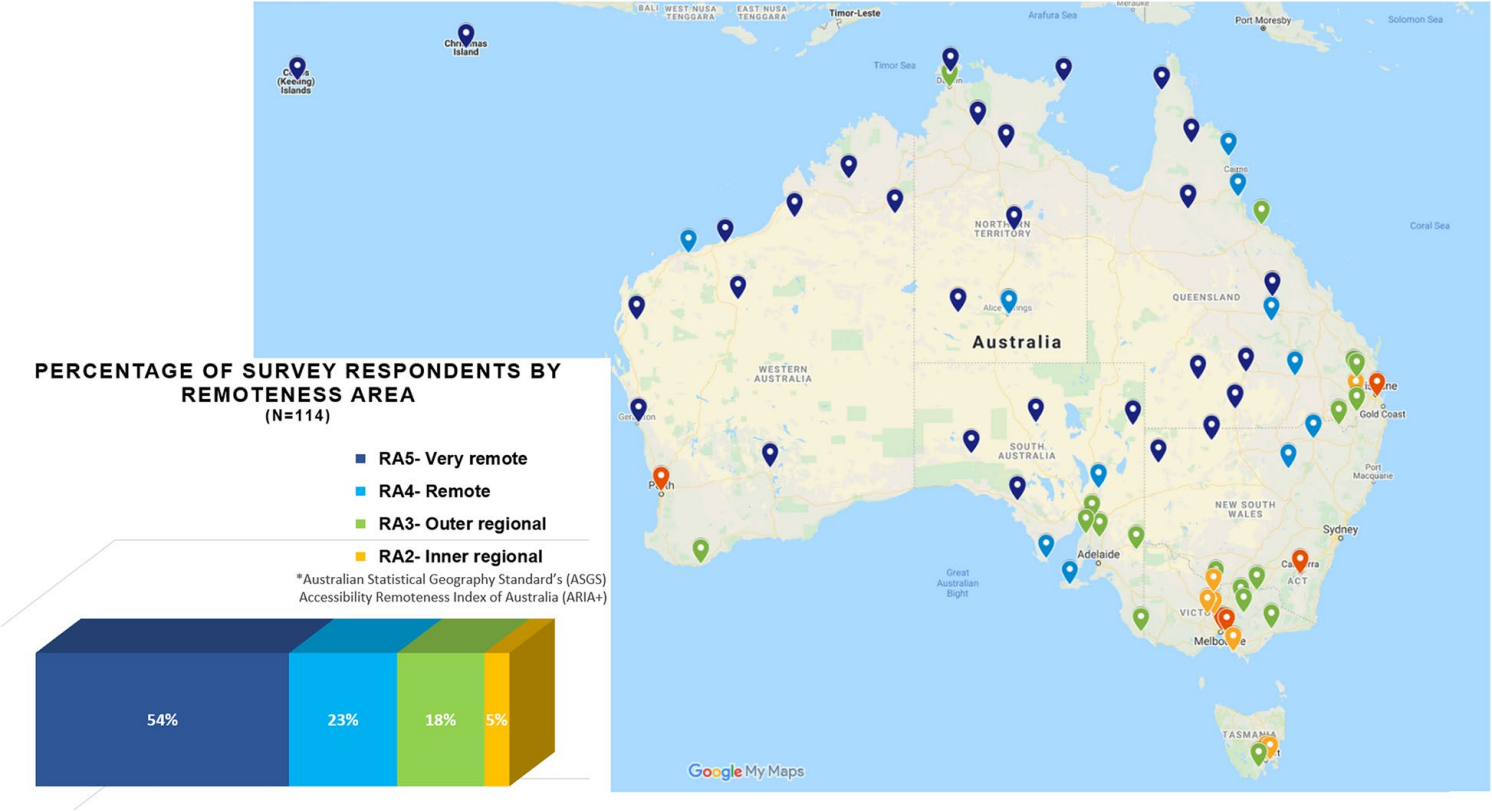
Survey respondents’ work locations by remoteness area *ASGS ARIA + 2016- The Australian Statistical Geography Standard (ASGS) Accessibility Remoteness Index of Australia (ARIA) defines 5 geographical categories or remoteness areas (RAs) determined by road distance from the closest urban centre [24, 25]

From a total of 120 responses received nationally, 114 valid responses were included in the analysis following raw data review by 3 researchers. The survey completion rate was 85%. Six respondents were excluded for supplying postcodes from Major city (RA1) areas; while these individuals may have worked in rural locations (RA2-RA5) in the past or currently in a locum/part-time capacity, this was not able to be reliably determined. Figure 1 shows the percentage of responses by ASGS ARIA + 2016 remoteness area [24, 25] and illustrates respondents’ clinic/work locations.

### Demographics of survey respondents

Registered midwives (RMs) accounted for the largest proportion of respondents by role (52/114) and, together with nurses (25/114), made up half of all survey respondents (17 dual qualifications- RM and nurse), followed by General Practitioners (GPs) (40/114, 6 dual qualifications- GP Obstetricians). The vast majority (86%) of responses were received from female clinicians (98/114), reflective of the female-dominated nursing and midwifery professions. Most respondents were experienced clinicians, with 90% (102/114) having more than six years of clinical practice in their fields. See Supplementary Table S4 for survey respondent characteristics (gender, role, experience and location/remoteness area).

### Ultrasound use during antenatal care at respondents’ clinics

**Fig. 2:**
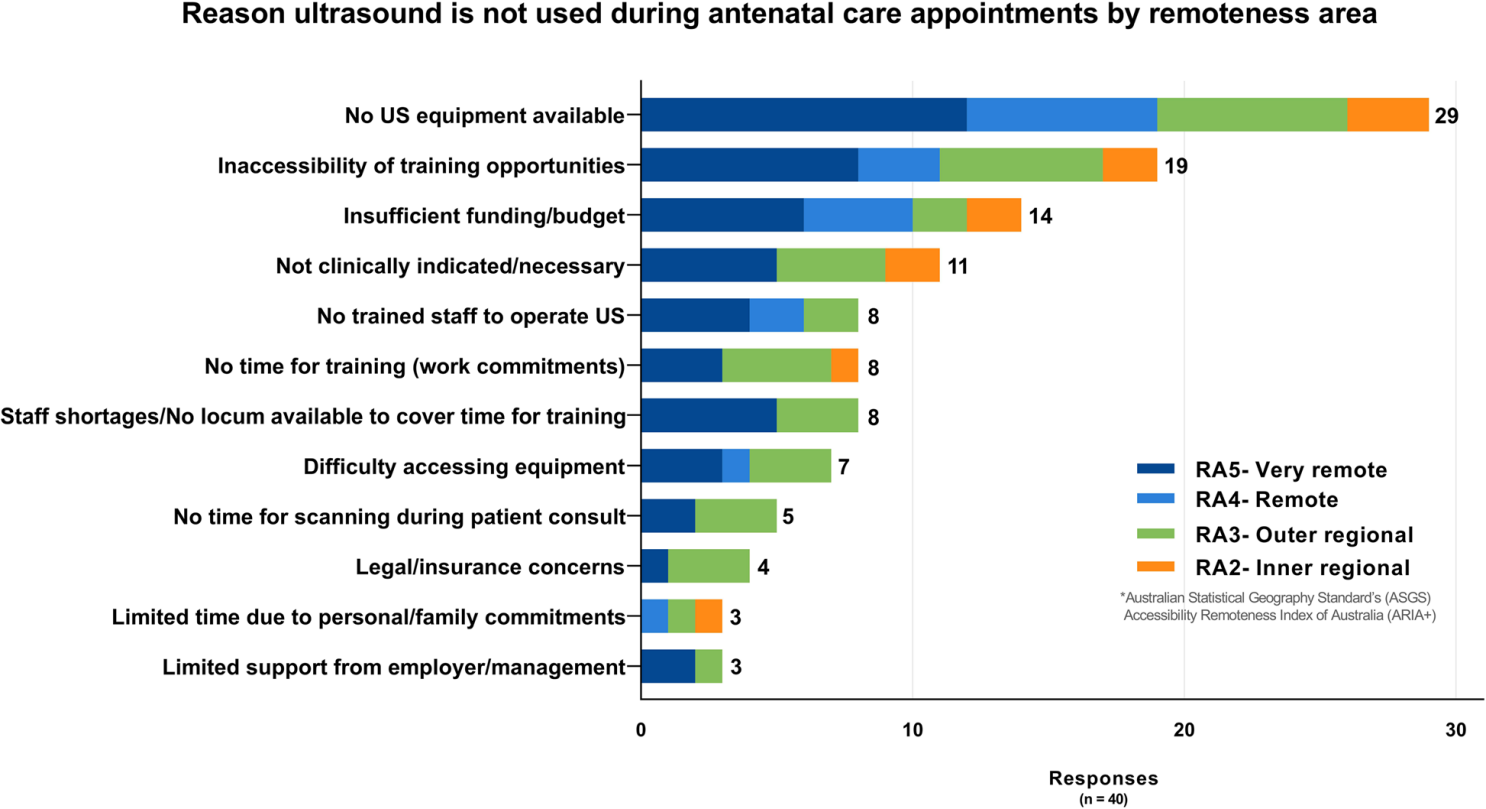
Why ultrasound is not used during antenatal care appointments by remoteness area *ASGS ARIA + 2016- The Australian Statistical Geography Standard (ASGS) Accessibility Remoteness Index of Australia (ARIA) defines 5 geographical categories or remoteness areas (RAs) determined by road distance from the closest urban centre [24, 25]

**Fig. 3:**
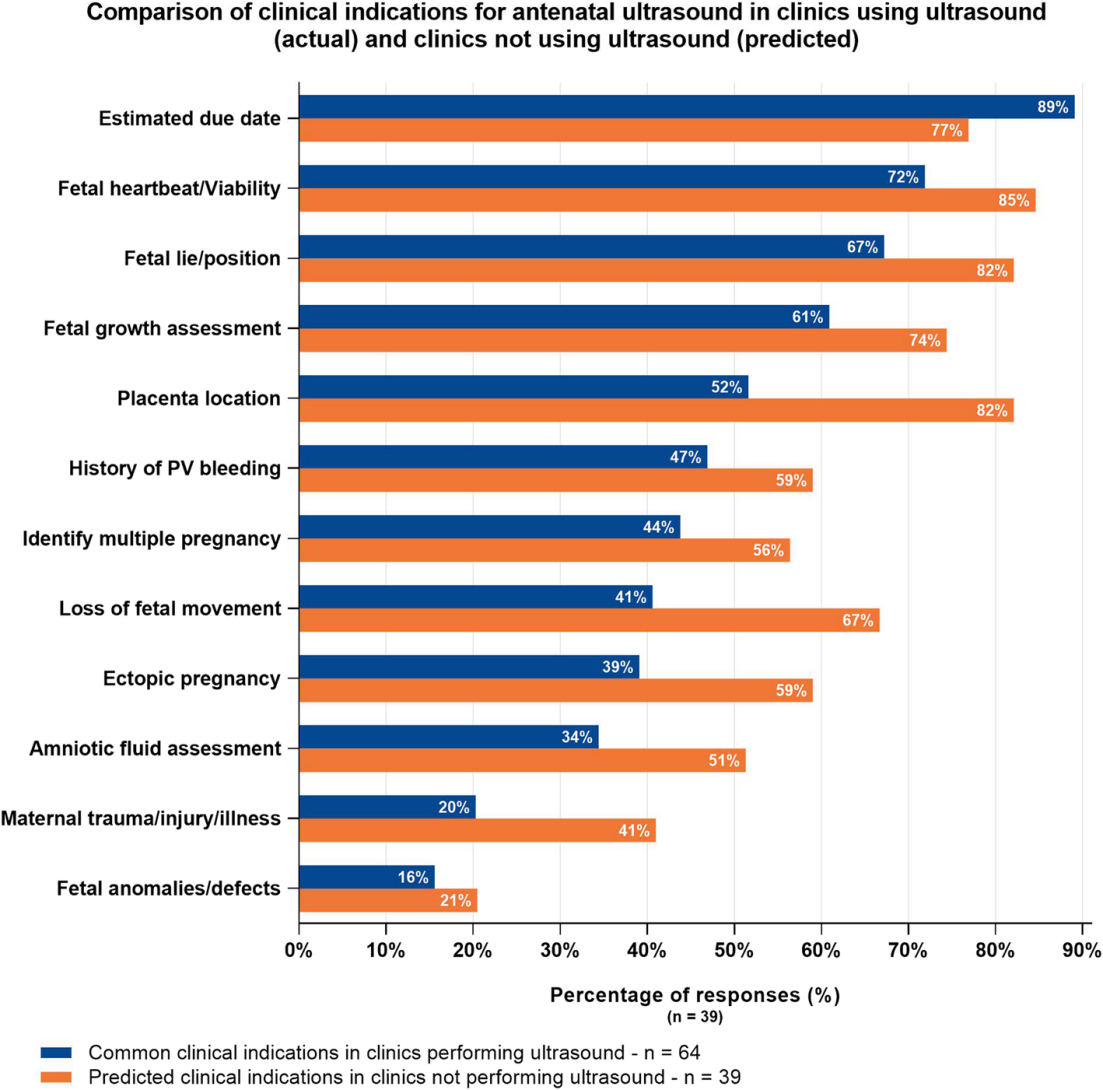
Comparison of clinical indications for antenatal ultrasound in clinics using ultrasound (actual) and clinics not using ultrasound (predicted)

**Fig. 4:**
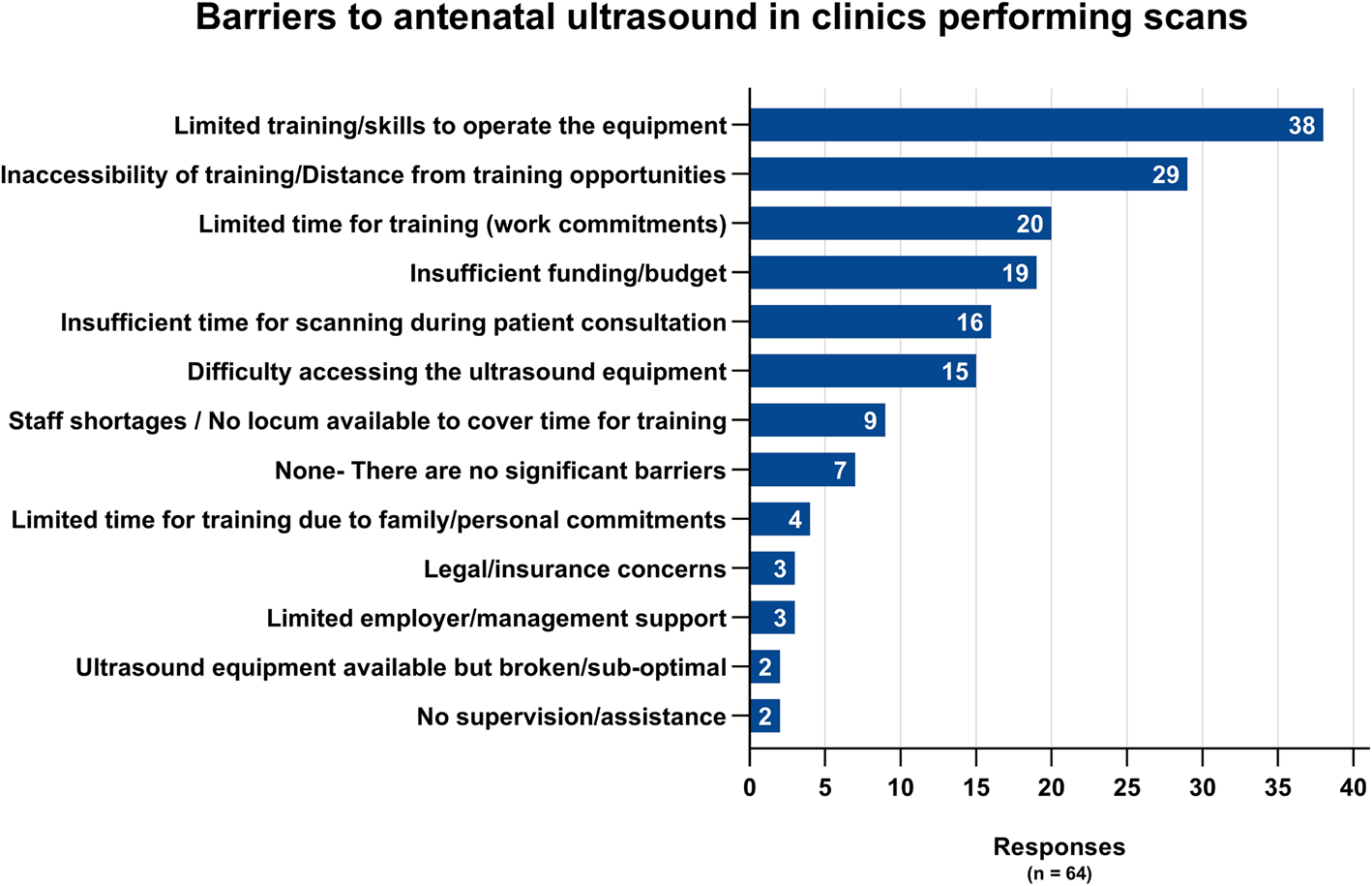
Barriers to ultrasound use in clinics performing ultrasound during antenatal care appointments

Of 111 responses received, 39% (43/111) reported that ultrasound was not used during antenatal care provision at their clinic. Lack of ultrasound equipment was the main reason provided (73%, 29/40), followed by inaccessibility of training opportunities (48%, 19/40) as shown in Fig. 2. Of the respondents reporting ultrasound was used at their clinics during antenatal care appointments (61%, 68/111), medical physicians (GPs and GP/Obstetricians) were performing the most PoCUS scans compared to other professional groups (58/62 for physicians and 49/62 midwives/nurses). Clinicians working onsite (as opposed to visiting clinicians) represented approximately two-thirds of those performing PoCUS scans (67%), and these were primarily carried out by registered midwives and general practitioners (see Supplementary Fig. S2). Only 14 respondents reported a sonographer performs ultrasound at their clinic: six onsite and eight visiting sonographers. In clinics using ultrasound, the main clinical indication was to estimate due date (89%, 57/64). Figure 3 shows the clinical indications in clinics performing ultrasound compared to the predicted use in clinics not performing ultrasound (i.e. if ultrasound became available). The main barriers to scanning reported by clinicians from clinics performing ultrasound were: Limited training/skills to operate the available equipment (59%, 38/64); Inaccessibility/distance from training opportunities (45%, 29/64) (see Fig. 4).

### Ultrasound equipment

Thirty-two per cent (33/104) of respondents overall reported having no ultrasound machine available at their clinic. Half of the respondents had a single ultrasound machine available at their clinic (49%, 51/104), but 28% of these were located offsite (brought in or loaned). Supplementary Figs. S3 and S4 show the number and location of ultrasound units available.

Portable ultrasound units (80%, 57/71) were four times more common than larger stand-alone ultrasound units (20%, 14/71). Almost half (42%, 25/59) of the ultrasound units described were older than five years. Fourteen per cent (8/59) were older than ten years and would not be recommended for clinical use (see Supplementary Fig. S5). Equipment malfunction/breakdown was reported by 23% (16/71) of respondents, 49% (35/71) had never experienced a breakdown, and 28% (20/71) were uncertain. Of the equipment that had broken down, almost half (7/16) reported weeks to months for repairs to be conducted (see Supplementary Fig. S6).

### Patient access to antenatal ultrasound

Of 102 respondents, 60% (61/102) reported pregnant women in their communities have no after-hours access to ultrasound, and of those that did (40%, 41/102), most were limited to basic bedside ultrasound provided by on-call staff, and only when that staff member was skilled in performing PoCUS. In other cases, hospital admission was required and only available in an emergency, and for some patients this required significant travel. Non-attendance at antenatal care appointments was reported to be a problem at 59% (59/100) of clinics. Some respondents stated their clinics regularly follow up non-attenders, making multiple re-bookings following missed appointments. Absenteeism was more prevalent in Remote (RA4) and Very remote (RA5) areas (64% of responses) compared to regional (RA2 and RA3) areas (39% of responses).

**Fig. 5:**
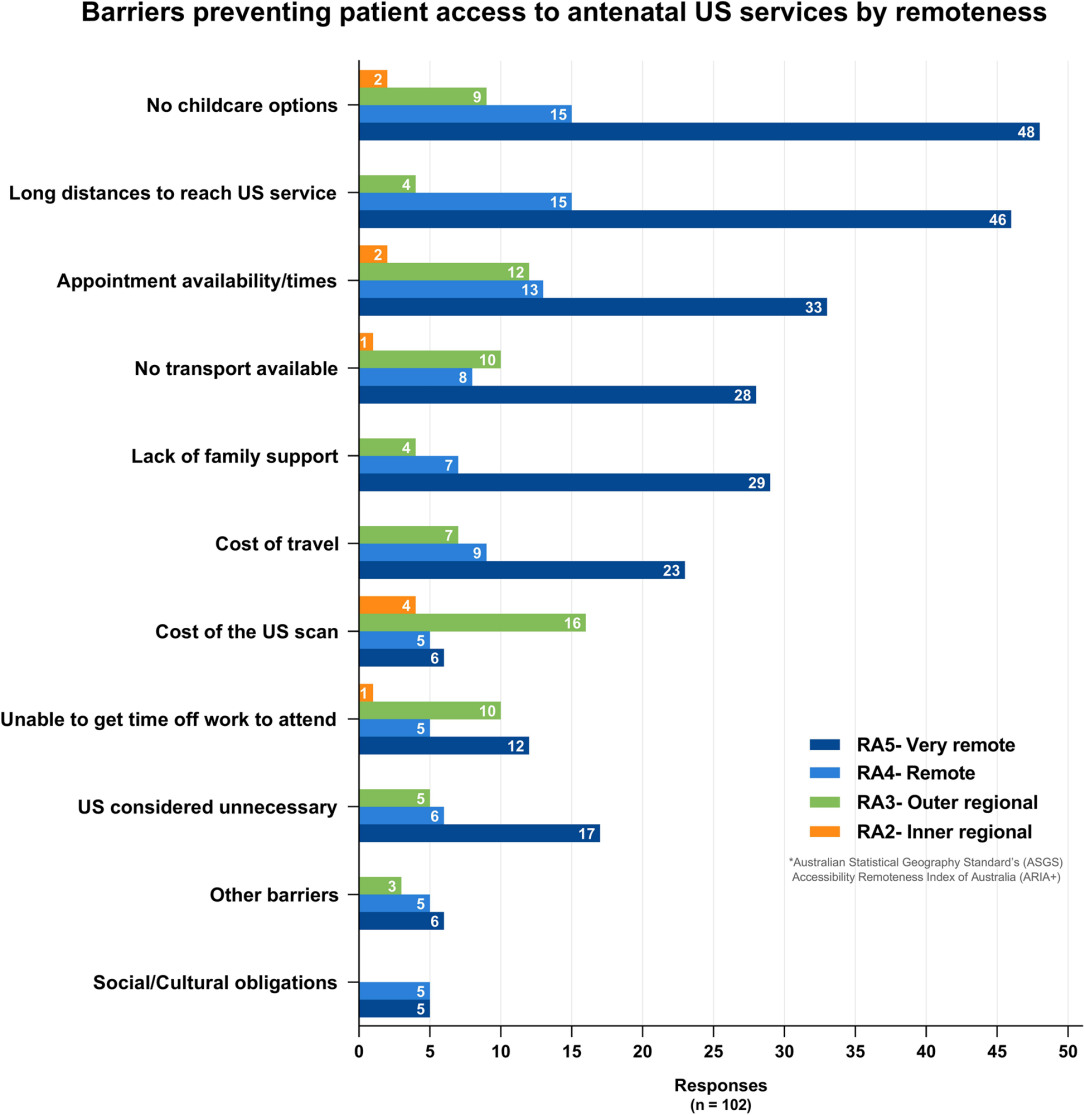
Barriers preventing patient access to antenatal ultrasound services by remoteness Note: Barriers reported are those perceived by the clinician/healthcare worker. *ASGS ARIA + 2016- The Australian Statistical Geography Standard (ASGS) Accessibility Remoteness Index of Australia (ARIA) defines 5 geographical categories or remoteness areas (RAs) determined by road distance from the closest urban centre [24, 25]

The main barriers, as perceived by the healthcare clinician, preventing women from accessing antenatal ultrasound services were lack of childcare options (73%, 74/102), long distances to reach ultrasound services (64%, 65/102), appointment availability/times (59%, 60/102), and having no available transport (46%, 47/102) (see Fig. 5). Other barriers (14%, 14/102) included: No accompanying/escort person permitted; Limited accommodation options; No bulk-billed scans available; Homelessness; Culturally inappropriate imaging service (only male sonographers); Sonographer shortage; Environmental conditions.

### Travel to reach ultrasound services

Over half (60%, 61/101) of the respondents had patients in their care who were required to travel out of their community (travel greater than 3 h) for ultrasound services, 41% (41/101) had patients travelling over 6 h, requiring overnight accommodation (“*If woman has to travel in by bus into nearest hospital for a scan, as is the case for the majority of women, it means 3–4 days away from family and home.”* Survey response) (see Supplementary Fig. S7 for travel time to reach ultrasound services). Forty-six per cent of respondents (47/102) named lack of available transport as a barrier to women accessing antenatal ultrasound services, with both travel and accommodation options reported to be limited or even non-existent. Several respondents reported patients forgoing ultrasound completely due to cost, distances and lack of travel and accommodation options (“*The clinic bus takes them, but we have had women not wanting to attend as they are away all day* [regardless of their appointment time] *and don’t have any money for food in town *etc*.”* Survey response). In some cases, buses were the only option but were not always operating (“*Bus only runs 2 days per week.”* Survey response) or appropriate for unwell, complex or heavily pregnant patients, particularly given the long waiting times and poor road conditions. In the case of island communities, flights or a ferry were the only means to access formal ultrasound services, and PoCUS access was dependent on available staff/current locum skill set. For some remote patients requiring air travel, a full plane was often required before charter and missed flights could mean the ultrasound is performed outside the optimal window for imaging *(“If they miss the flight to Cairns they have to wait 10 days to rebook.*” Survey response) (see Supplementary Fig. S8 for mode of transport used to reach ultrasound services).

Half of all respondents (50%, 57/102) reported limitations to existing travel arrangements at their clinics, with 20% (19/96) stating travel assistance was unavailable to pregnant women in their community. Of those who did have travel assistance (80%, 77/96), limitations and barriers were described (see Supplementary Table S5). Various government-funded patient travel assistance schemes were mentioned by half, some emphasising the limitations of these schemes, citing strict policy/eligibility criteria. Half (54%, 33/102) of the respondents reported no funding for an accompanying person to travel with the pregnant patient for ultrasound imaging, and in some cases (clinic-provided transport), this was not an option regardless of funding. The absence of an accompanying person was raised in qualitative responses as a barrier to women attending scanning services.

### Perceptions and attitudes of the healthcare worker to antenatal ultrasound

When asked if they thought Antenatal ultrasound is essential to prenatal care, 96% (96/100) of respondents either strongly agreed or agreed. The importance of ultrasound in antenatal care planning and decision making particularly for identifying high-risk patients and accurate gestational dating was stressed in qualitative responses (19/38). Other themes included the importance of antenatal ultrasound in remote settings (9/38), reassuring patients and decreasing maternal anxiety (3/38), usefulness in patient education (3/38) and its role in routine antenatal care (3/38). Four (4/100) respondents remained neutral, with explanations by two surrounding: ultrasound not being considered essential in low-risk pregnancies with certain conception dates; risk of overutilisation; poor pre-scan patient information; unsolicited gender reveal; and inconclusive findings causing patient anxiety.

Perceived increased interest (“[patients] *asking more questions*”), maternal engagement and bonding were reported following visualisation of the baby and some patients expressed appreciation for having the scan performed locally (PoCUS). Most respondents (77%) felt antenatal ultrasound impacted positively on a patient's lifestyle choices and behaviour (e.g. exercise, diet, reduction/cessation of smoking/drinking). Supplementary Table S6 lists qualitative responses regarding clinicians perceived impacts of ultrasound on patient lifestyle, and Supplementary Table S7 provides a sample of responses. Only one respondent (1/100) felt ultrasound could also produce a negative behavioural response, stating reassurance provided by a ‘normal’ ultrasound can reinforce existing harmful behaviours and the stress of identified complications can lead to poor lifestyle choices.

Sixteen per cent (16/100) of respondents reported patient refusal of antenatal ultrasound scans. The most common reason for refusal was a perceived lack of benefit from the scan or the patient considered it unnecessary, followed by the costs involved (scan and travel expense), concerns regarding harm to the fetus, and having to travel out of their community. Table 1 lists the reasons provided to clinicians in order of frequency with sample responses.

**Table 1:** Reasons provided to clinicians by pregnant women refusing to have an antenatal ultrasound scan

Theme	^a^Frequency of theme	Sample responses
Deemed unnecessary/Lack of perceived benefit	5	*“Patient cannot afford travel or believes the scan unnecessary.”*
Cost of the scan and/or travel	3	*“Even if medically indicated cost has been an issue at our clinic.”*
Concerns regarding ultrasound effect on fetus/Desire for minimal medicalisation of antenatal care	3	*“Concerns regarding long-term effects on fetal development, scared findings will result in over medicalisation of pregnancy journey.”*
Having to travel out of the community	3	*“Mainly due to it being away from town [i.e. out of the patient’s local community].”* *“Our ladies have so much further to travel to access services that are available to the women who live in cities.”*
No childcare options/Worry over leaving other children for long periods	2	*“The main reason is family concerns, having to leave their children for long periods of time and worried children may be unsafe.” “[having to travel] presents major problems with Mums who already have little ones at home and become very stressed and upset about having to leave their families to travel so far away.”*
Afraid to attend scan alone	1	*“Only if no one is available to mind children or if frightened to go alone.”*
Religious or spiritual beliefs	1	*“Occurs rarely, but usually around religious or spiritual beliefs.”*

^a^Frequency of theme taken from qualitative free-text responses. Reason for refusal reported by the healthcare worker

### Training and Continuous Professional Development (CPD)

**Fig. 6:**
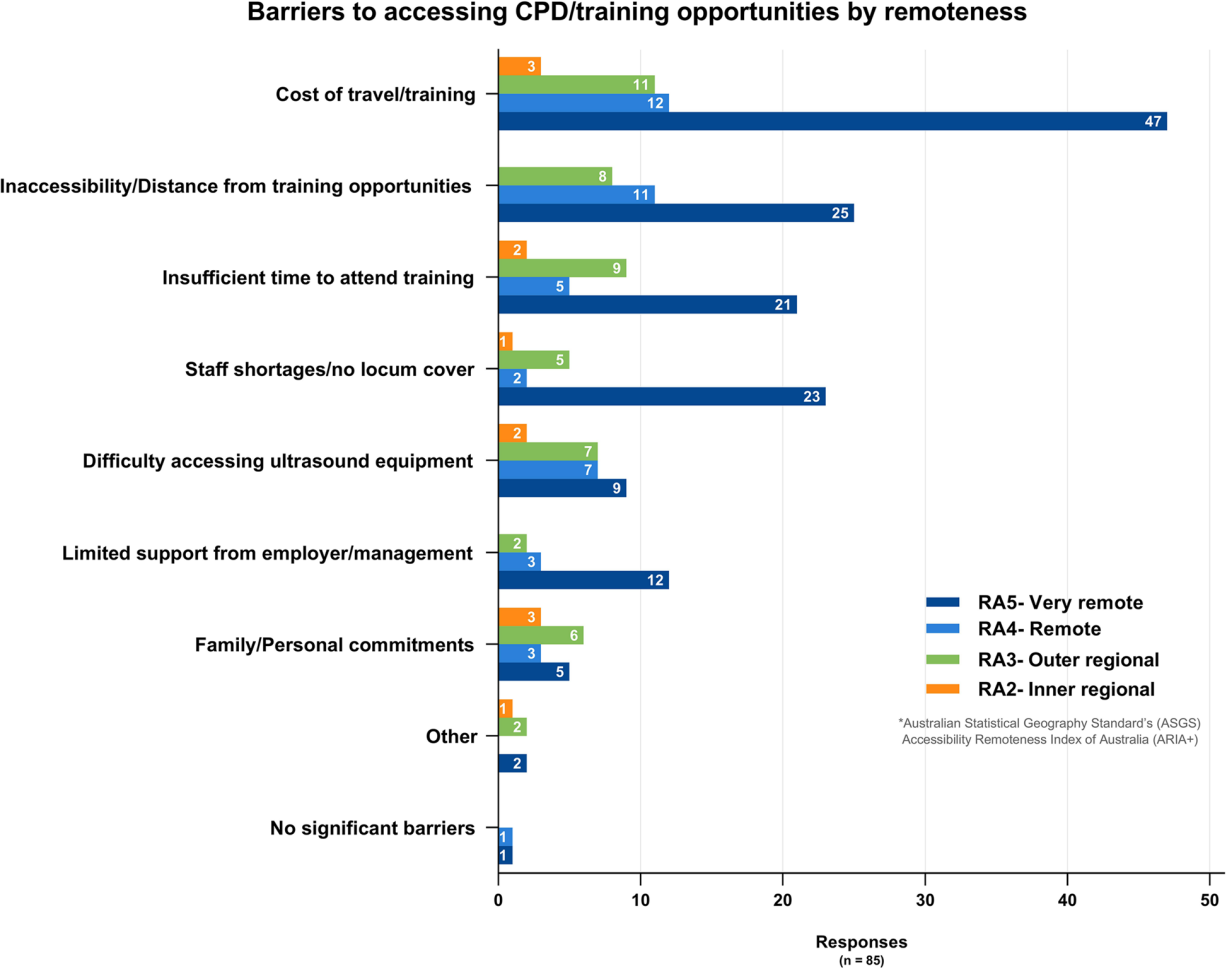
Barriers to accessing CPD/training opportunities by remoteness area *ASGS ARIA + 2016- The Australian Statistical Geography Standard (ASGS) Accessibility Remoteness Index of Australia (ARIA) defines 5 geographical categories or remoteness areas (RAs) determined by road distance from the closest urban centre [24, 25]

The majority of respondents (87%, 87/100) indicated they would be interested in undertaking training in basic antenatal ultrasound. The main barriers to accessing training provided by these respondents were: The cost of training and travel (86%, 73/85), and Inaccessibility/distance from training opportunities (52%, 44/85). Figure 6 shows reported barriers to ultrasound training opportunities by remoteness area. Free text responses regarding ‘What would make it easier for you to learn/increase your ultrasound skills?’ cited better access to training (65%, 55/85), and access to ultrasound experts (21%, 18/85) for onsite supervision, scan/image review and ongoing mentoring/support. Other recurrent themes surrounded ultrasound equipment accessibility, time and opportunity to practice, financial support (for courses and travel), and locum support to cover attendance at training. Thirteen per cent (13/100) of respondents indicated they were not interested in undertaking basic training in antenatal ultrasound, most (9/13) stating they already possessed a basic ultrasound skill set (see Supplementary Fig. S9). Three respondents (3/13, 2 RMs, 1 GP) felt ultrasound was an unnecessary skill for them or had insufficient antenatal patient load to justify training.

At the survey’s conclusion, 36 respondents made an optional final comment regarding the provision of antenatal ultrasound services in rural Australia. These responses are provided in Supplementary Table S8.

## Discussion and recommendations

This study’s aim to explore the status of antenatal ultrasound availability and use in rural Australia was achieved through a survey canvasing RA2, RA3, RA4 and RA5 (ASGS ARIA + , 2016) [24, 25] healthcare clinicians regarding: Use of antenatal ultrasound in their rural clinics, patient access to antenatal ultrasound within their community, and access to and interest in continuing professional development/training opportunities. The two main obstacles to providing antenatal ultrasound services in rural Australia identified in this survey were lack of trained staff (including access to training opportunities) and inaccessibility of ultrasound equipment. Thirty-nine per cent of respondents reported antenatal ultrasound was not used at their clinic and one-third had no access to an ultrasound machine. Geographical isolation and distances required to travel present a significant barrier to clinicians accessing training opportunities and pregnant women accessing healthcare and ultrasound services.

### Upskilling rural healthcare clinicians

Basic antenatal ultrasound skills can be effectively taught to medical and allied health professionals through intensive short courses [27–32]. Despite facing barriers to access, this survey suggests many rural clinicians are interested in undertaking training to increase their skill-set and provide improved antenatal care and services to patients. Responses indicated that some rural clinicians are unaware of the training courses available to them; that they can advance their scope of practice to include ultrasound with or without formal accreditation *(“Limited appropriate courses—most courses are very basic or are aimed at sonographers, e.g. DDU.”, “Not a course I had previously thought was available to nurses/midwives.”* Survey responses). Promoting the existence and availability of PoCUS courses and accreditation pathways could increase the number of rural clinicians pursuing training to advance their clinical skills.

Ultrasound training is seeing greater integration in undergraduate medical curricula and on-the-job training, but it is less established in nursing and midwifery programs [30, 33–35]. Our data reflected this trend, with medical practitioners (GPs and GP/OBGYNs) reported to be performing PoCUS more than other professional groups and made up the majority of those not interested in training due to already possessing a basic ultrasound skill-set. In many low-resource settings, antenatal care is provided primarily by midwives and nursing staff. Within Australia, nurses and midwives are the only professional healthcare groups represented in greater numbers in Remote (RA4) and Very remote (RA5) areas compared to medical practitioners and allied health professionals [36] (see Supplementary Fig. S10), presenting an opportunity to upskill and task-shift to these essential workers. Where sonographers are unavailable, broadening the scope of practice for nurses and midwives (along with GPs/doctors) to include basic antenatal ultrasound services, with appropriate training and clear practice definitions, would increase workforce capacity in these under-resourced areas. Expanding roles and scope of practice can also help to empower health professionals leading to greater job satisfaction and rural workforce retention [37, 38].

Inaccessibility/distance from training courses, cost of travel, insufficient time (family and work commitments) and staff shortages were provided as significant barriers to training access in this survey. Subsidising ultrasound training schemes for rural clinicians to cover course and travel costs could help incentivise clinicians towards upskilling. However, given most available face-to-face PoCUS courses are city-based, such subsidies would not address the considerable time commitment required for travel for course attendance. Providing local/rural-based training programs would overcome the need for frontline rural healthcare clinicians to leave work and family commitments, and for locum staff replacements to cover their absences while travelling and attending urban training centres. This may also prove a more cost-effective approach to training and warrants further evaluation. Given the broad scope of skills required by rural generalists, training programs delivered locally would also minimise the need to prioritise particular courses or skills over other clinical areas due to inaccessibility of local courses ( *“I can only access off-site training once a year. With many portfolios it is difficult to prioritise only one each year.”* Survey response).

Access to clinical supervision and mentorship is an ongoing challenge for rural clinicians. Many, particularly those working in remote communities and performing community visits, practice in isolation and struggle with poor telecommunications access [37]. Respondents to this survey highlighted a need for better access to ultrasound experts for onsite supervision, ongoing mentoring/support and to review/discuss patient images. The ASUM accreditation pathway provides clinicians with access to skilled ultrasound professionals to provide support, mentorship and image review. Increasing access to ASUM’s PoCUS accreditation pathways formally certifying clinicians’ skills would assist in consolidating clinical competency and provide clinicians with additional distance support. However, review of accreditation requirements is warranted given the difficulty rural clinicians face performing formative and summative supervised practice assessments. ASUM allows half of these assessments to be completed in a non-clinical environment, but under direct expert supervision with feedback provided. Supervision and assessment through Telehealth and Teleultrasound may offer a solution to reduce travel requirements for rural clinicians interested in training and formal certification [39].

While most clinics (80%) had Telehealth available, some respondents were unclear on what it was used for or how it could be applied to ultrasound. Limitations cited included: Technical issues *(“NBN too slow”, “sound delay”, “technology not good enough”*); Logistics of setting-up/running a Telehealth system (*"access to clinical experts, Telehealth system in an appropriate environment for private consult"*); and ultrasound being a *"hands-on user dependent modality requiring an expert to operate the probe"*. A clinic manager stated Telehealth was “*too labour intensive in remote health*”, despite it being the only option for access to medical specialists for some remote patients [10]. Education campaigns on the advantages of Telehealth/Teleultrasound for education, post-training support, clinical supervision and access to specialists/experts could assist uptake of the technology by rural clinics/clinicians. Given the technical obstacles evident in many rural and remote locations [22, 40], government support to assist rural clinics in implementing telehealth systems, and improving telecommunication infrastructure is indicated. Difficulty accessing centralised patient medical records and imaging/scans and reports required for comparison with PoCUS scans (both good practice and a requirement for ASUM logbook records) was also noted in survey responses *(“*[Barriers include a] *lack of access to digital records.”* Survey response). Access to centralised medical repositories like the government’s ‘My Health Record’ could assist rural clinicians in this regard.

The lack of remuneration for training, skills, equipment and time scanning patients was reported in this survey (“*Lack of Medicare funding stops some practices from increasing ultrasound use.”, “Medicare rebates would be helpful to encourage practices to start using ultrasound.”* Survey response) and in a recent study by Arnold et al. (2023) [39]. Establishing and simplifying access to financial incentives with Medicare for remote PoCUS use could encourage clinicians to purchase equipment, undertake training and take time to scan patients during consults. Notably, *Medicare billing requires a patient record with written report to be generated and retained (“No Medicare Benefits Schedule item numbers to claim for time, skills and equipment use without a certificate and providing a report”* Survey respondent), which can present an additional barrier to establishing and maintaining a subsidised ultrasound service [12, 39, 41, 42].

### Ultrasound equipment

Modern portable ultrasound machines capable of producing high-quality images [43, 44] can be purchased for around $40,000 (AUD). These units offer the advantage of being easily transported by clinicians between clinics and to community/outreach/home visits. In cases where patients are unwilling or unable to travel significant distances, this may provide clinicians with the only opportunity to image the fetus to acquire accurate gestational dating, identify high-risk pregnancies, and establish placental and fetal position before birth. In rural clinics with staff trained in the use of PoCUS but no available equipment, a relatively small initial expenditure supplying equipment could provide substantial benefits, assisting in antenatal care planning and referral, reducing unplanned out-of-hospital births and unnecessary and costly travel for some patients.

Within this survey, the most frequently provided reason for not using ultrasound during antenatal care appointments was the inaccessibility of ultrasound equipment. Overall, one-third of respondents had no access to an ultrasound machine. Half had access to a single ultrasound unit but these were often shared between staff and other clinics within the area health service requiring advanced booking. In one case, an ultrasound was shared between 6 clinical sites. Large stand-alone ultrasound units made up 1/5 of the ultrasounds described. The provision of portable ultrasound units would allow clinicians to travel with the equipment and provide opportunistic scanning at community/home/outreach visits. The use of ultrasound in low-resource settings has been shown to increase attendance at antenatal care appointments and parental engagement with pregnancy care [45–47], and was described by respondents to this survey. Having the capacity to offer PoCUS directly to patients in remote communities would enable clinicians to access, engage, and provide timely referrals to a population of women who would otherwise receive limited or no antenatal imaging. However, providing equipment to clinics in need will not improve service delivery and could represent a risk to patients if staff have not received appropriate training in its use. In such cases, the provision of combined equipment and subsidised training is warranted.

The quality and age of available imaging equipment in rural areas was a concern raised by the Senate’s 2018 report [8], and was reflected in this survey. Equipment older than ten years is at risk of breakdown and can be difficult and expensive to repair. Image quality can be degraded and far from the resolution achievable by newer state-of-the-art equipment. Ideally, replacement and renewal of ultrasound equipment should be carried out every 5–10 years [48], and formal replacement programs may be indicated.

### Patient access

Lack of childcare options for other children at home, long distances to reach services (often without escort), lack of appointment and travel options, and costs associated with travel and scans were identified by healthcare clinicians in this survey as barriers to rural women accessing ultrasound services. Travel options to reach ultrasound services are, for some rural and remote women, limited, non-existent, or can come at significant additional cost, particularly where distance necessitates air travel or overnight accommodation (“*Accommodation when coming to Ceduna can be limited and costly and no one wants to pay for it.”* Survey response). Additional costs incurred by rural patients accessing services was highlighted by the Senate’s 2018 enquiry [8], with the cost of scans and travel cited in this survey as barriers to women accessing antenatal ultrasound imaging. One respondent from a remote island community stated they feel strongly that *“rural women are disadvantaged in paying heavily for scans in pregnancy at private imaging services. It is like a monopoly—the women have no choice. This is wrong and should be subsidised I believe”.* Another respondent reported “*Some ladies won't/can't have the ultrasounds if they have to pay because they just don't have the money”.* Review of scan costs (patients’ out-of-pocket costs) and Medicare rebates for rural patients is advised.

Travel and accommodation costs can far exceed scan costs depending on distance, mode of transport and need for accommodation. Fearnley et al. (2016) [49] quantified the costs of attending health care appointments, including travel, accommodation, wage loss and unwaged time loss and found “Rural people must make a significant financial investment to access their ‘free’ health care”, presenting a significant barrier particularly for those suffering socioeconomic deprivation. While some form of travel assistance was reported to be available to most respondents’ patients in this survey, limitations were described (e.g. available to complex pregnancy, breastfeeding or Aboriginal women only; capped prices; cheapest travel option only which may be inappropriate for unwell/heavily pregnant patients; closest or Patient Assistance Transport Scheme determined radiology service only; specific or specialist ordered scans only; ineligibility if missed previous appointments). Various government patient travel assistance schemes operate throughout Australia, each with its own policies and eligibility criteria [50–52]. The need to travel alone was cited regularly as an impediment to women attending appointments and a limitation of some of these schemes. Pregnancy can be stressful, particularly for first-time mothers and those experiencing complications or having experienced poor outcomes previously. The absence of a support person while travelling and during antenatal care and imaging appointments can make what should be an exciting time, a significant ordeal, particularly for Aboriginal women. Existing patient transport schemes would benefit from review to: increase subsidies to cover more of the travel costs; make the schemes more visible to patients and staff; simplify application processes; broaden eligibility criteria making them more accessible; and provide allowances for an escort (for schemes that limit this option).

### Attendance at appointments

Research has shown ultrasound use when providing antenatal care can reassure patients, build Clinician-Patient rapport, improve the perception of service quality and increase patient attendance [53–56]. Similarly, respondents to this survey reported ultrasound use in their clinic increased antenatal care attendance and compliance with care recommendations, reassured patients, fostered maternal and paternal bonding with the baby, and improved maternal lifestyle choices. Reassurance over fetal well-being was the main reason reported for patient attendance at ultrasound appointments. Absenteeism from antenatal care appointments was described. Socioeconomic barriers, cultural obligations and regular travel between communities were cited in qualitative responses as perceived reasons for lower antenatal care and ultrasound attendance in more remote clinics and Aboriginal populations (RA4 and RA5 responses). This is reflective of Smith et al. (2008) findings that rurality largely impacts health outcomes by exacerbating the effects of “*socioeconomic disadvantage, ethnicity and poorer access to health services, compounded by higher levels of personal risk and more difficult environmental, occupational and transportation conditions*” [57]. Statistics from 2019 show the proportion of Aboriginal mothers attending first-trimester antenatal care was 8% lower than for non-Indigenous mothers, with those in Remote and Very remote areas most likely to receive no care or first attend after 20 weeks [6]. Within this survey, one clinic reported operating a drop-in (no appointment required) service, which could simplify and encourage attendance, particularly for Aboriginal patients. Additionally, outreach/home/community visits by trained clinicians with portable ultrasound equipment could greatly benefit remote and Aboriginal populations. Rural communities may also benefit from targeted, culturally sensitive education campaigns to increase patient awareness of the benefits of antenatal care and ultrasound early in pregnancy.

Cultural awareness underpinning health professionals’ practice is crucial to providing culturally appropriate maternity services and improving pregnancy outcomes for Aboriginal mothers and their babies [58]. Unfortunately, Australian maternity healthcare centres largely reflect Western medical values and perceptions of health. (“*Indigenous women hate waiting in strange hospital environments, are often found outside and can be assumed missing or not attending when called*.” Survey response). It was evident in this survey that cultural awareness and sensitivity were lacking in a small minority of survey responses. This and the poorer health outcomes experienced by Aboriginal mothers and babies indicate greater focus on providing culturally sensitive care to women in rural communities is needed, ideally through increasing the number of Aboriginal healthcare workers in these regions and targeting these workers for PoCUS training. Additionally, education initiatives teaching cultural sensitivity should be implemented or reviewed for the existing and future rural workforce.

### Implications of this study

Antenatal PoCUS can be a powerful tool in the hands of appropriately trained rural clinicians and can provide considerable benefits to patients with limited/no access to ultrasound services (“*If issues are picked up earlier for the ladies it isn't so traumatic for them, and if they don't have to travel so far for an ultrasound and pay so much money there will be a lot less stress experienced*.” Survey response). Training rural clinicians in PoCUS has the potential to save healthcare dollars [59, 60] and provide considerable reassurance to pregnant women while reducing the need to travel and time away from family). This novel study provides a view of the status of antenatal ultrasound service use and availability in rural Australia and explores barriers faced by rural health professionals and their patients. Perspectives are from experienced frontline multi-disciplinary healthcare professionals responsible for planning and providing antenatal care in rural areas. This has informed recommendations and strategies (Supplementary Table S9) that could, through a coordinated approach from researchers, clinicians, policymakers, educators and health services, combat service inequality to better meet the needs of pregnant women and babies living in disadvantaged communities. While this study was aimed at Australian clinicians, parallels in service access and health outcomes in low-resource settings of other developed and developing counties [61, 62] may allow some findings and recommendations to be applied globally.

Future research efforts into the viability of antenatal PoCUS should be directed towards studying direct clinical outcomes and cost-benefits in the rural setting to inform implementation initiatives aimed at establishing this valuable resource into standard practice for rural clinicians. Further studies of patient experience [56] and perspectives surrounding antenatal ultrasound access in rural communities would provide critical insight into barriers faced and complement the perspectives drawn from healthcare workers surveyed in this study.

### Limitations of this study

This survey was distributed to health professionals only. Views/opinions on patients accessing services are taken from the healthcare professionals’ observations and experiences, limiting the validity of these questions. The small number of survey participants limits study power. Response rate could not be reliably established due to open advertising and tiered distribution methods used. The survey design provided respondents with anonymity for unbiased response, but prohibited follow-up on concepts or incomplete responses. As the survey was voluntary and not evenly distributed across all area health services (as in an audit), an accurate depiction of ultrasound equipment distribution could not be established. A series of analyses using responses from single postcodes was performed (sensitivity analysis) to account for multiple participant response and multiple responses from within a single clinic. The non-probability sampling method used could introduce bias and impact the generalisability of findings. Multiple survey reminders, broad advertising across professional disciplines, and an incentive (prize draw for training or conference attendance) were used to reduce non-response bias.

## Conclusion

Despite efforts to close the gap between rural and metropolitan health, variation in medical outcomes and access to services for women and babies persists. That this inequity compounds other issues related to race and social determinants of health is even more concerning. Future policy and efforts to combat inequitable service access must adopt a coordinated approach to meet the needs of pregnant women in rural Australia. This study suggests the provision of portable ultrasound machines, training in antenatal PoCUS with ongoing support/mentoring and accreditation of health professionals, and investment in telehealth infrastructure could strengthen rural workforce capacity. This, along with addressing the complex economic, environmental and socio-cultural barriers to accessing services faced by rural patients could help to improve antenatal care and pregnancy outcomes in these disadvantaged communities.

## Definitions

*Clinician*- a healthcare provider (e.g. nurse, midwife, doctor) working clinically.

*Physician*- a clinician who is a medical doctor (e.g. general practitioner, specialist OBGYN) who has completed a medical degree.

*ASGS ARIA* + *2016*- The Australian Statistical Geography Standard (ASGS) Accessibility Remoteness Index of Australia (ARIA +) defines five geographical categories or remoteness areas (RAs) determined by road distance from the closest urban centre. These categories aim to capture the ease or difficulty Australians face accessing services in non-metropolitan areas [25].

ARIA, created as a joint project between the Hugo Centre and the Australian Department of Health and Ageing in 1998, is the predecessor of the more widely used ARIA + , and continues to be developed and maintained by the Hugo Centre. ARIA + (2016 being the most recent data release) is a continuous varying index with values ranging from 0 (high accessibility) to 15 (high remoteness), based on road distance measurements from over 12,000 populated localities to the nearest service centres in five categories based on population size [24].

Rural- The term ‘rural’ within this report refers to areas outside Major cities (RA1), i.e. RA2 to RA5 of the ASGS ARIA + (2016) locations/categories. The term ‘remote’ encompasses more isolated areas, i.e. RA4 and RA5 regions.

The geographical distribution of Australia’s Remoteness areas is illustrated in Fig. S1 of Supplementary material (Fig. S1: Map of the Australian Statistical Geography Standard (ASGS) Accessibility Remoteness Index of Australia (ARIA) 2016 Remoteness Areas Australia [26]).

### Supplementary Information

**Additional file 1:** **Figure. S1.** Map of the Australian Statistical Geography Standard (ASGS) Accessibility Remoteness Index of Australia (ARIA) 2016 Remoteness Areas Australia  [26].*ASGS ARIA+ 2016 defines 5 geographical categories or remoteness areas (RAs) determined by road distance from the closest urban centre [24, 25].

**Additional file 2: Figure S2.** Health professionals performing ultrasound during antenatal care appointments at respondents' clinics.

**Additional file 3: Figure S3.** Ultrasound units available for use at respondents' clinics.

**Additional file 4: Figure S4.** Location of ultrasound units used at respondents' clinics. Note- Answer relevant to the main ultrasound where multiple units were available.

**Additional file 5: Figure S5.** Age of equipment used in respondents' clinics. Note- Answer relevant to the main ultrasound machine where multiple units were available.

**Additional file 6: Figure S6.** Time to repair damaged/broken ultrasound units at respondents' clinics. Note- Answer relevant to the main ultrasound machine where multiple units were available.

**Additional file 7: Figure S7.** Travel time required by patients to reach ultrasound services.

**Additional file 8: Figure S8.** Mode of transport used to reached ultraound services by respondents patients.

**Additional file 9: Figure S9.** Reasons provided for why respondents were not interested in antenatal ultrasound training.

**Additional file 10: Figure S10.** Number of practising healthcare workers per 100,000 population by profession and remoteness (2020) [36].

**Additional file 11: Table S1.** Perinatal, stillbirth and neonatal death rates per 1,000 births by Indigenous status and remoteness area (2020) [4]. **Table S2.** Maternal mortality ratio per 100,000 women giving birth by Indigenous status and remoteness area (2012-2020) [5].

**Additional file 12: Table S3.** Schedule of recommended antenatal ultrasound, components of screening and pregnancy complications.

**Additional file 13: Table S4.** Characteristics of survey respondents.

**Additional file 14: Table S5.** Limitations to existing travel arrangements at respondents’ clinics.

**Additional file 15: Table S6.** Impacts of ultrasound on patient lifestyle reported/perceived by respondents/clinicians.

**Additional file 16: Table S7.** Sample responses of ultrasound impact on patient lifestyle choices/behaviour as reported/perceived by respondents/clinicians.

**Additional file 17: Table S8.** Additional comments from survey participants regarding the provision of ultrasound services to pregnant women in rural Australia.

**Additional file 18: Table S9.** Recommendations from national survey.

**Additional file 19.** Needs Analysis Survey pro forma.

## Data Availability

The datasets used and/or analysed during the current study are available from the corresponding author upon reasonable request.

## Abbreviations

ARIA: Accessibility Remoteness Index of Australia
ASGS: Australian Statistical Geography Standard
ASUM: Australasian Society for Ultrasound in Medicine
CPD: Continuing Professional Development
FTE: Full-time equivalent
GP: General Practitioner
OBs: Obstetrics
OBGYN: Obstetrics and gynaecology
PoCUS: Point-of-Care Ultrasound
RA: Remoteness Area
RACGP: Royal Australian College of General Practitioners
RM: Registered midwife
