# Supplementary material for: Antenatal ultrasound needs-analysis survey of Australian rural/remote healthcare clinicians: recommendations for improved service quality and access
Source: BMC Public Health. 2023 Nov 17;23:2268. doi: 10.1186/s12889-023-17106-4 (PMC10655468; doi:10.1186/s12889-023-17106-4)
Supplement: Supplementary file 1 — Additional file 1: Figure. S1. Map of the Australian Statistical Geography Standard (ASGS) Accessibility Remoteness Index of Australia (ARIA) 2016 Remoteness Areas Australia [26].*ASGS ARIA+ 2016 defines 5 geographical categories or remoteness areas (RAs) determined by road distance from the closest urban centre [24, 25]. [file 12889_2023_17106_MOESM1_ESM.docx]

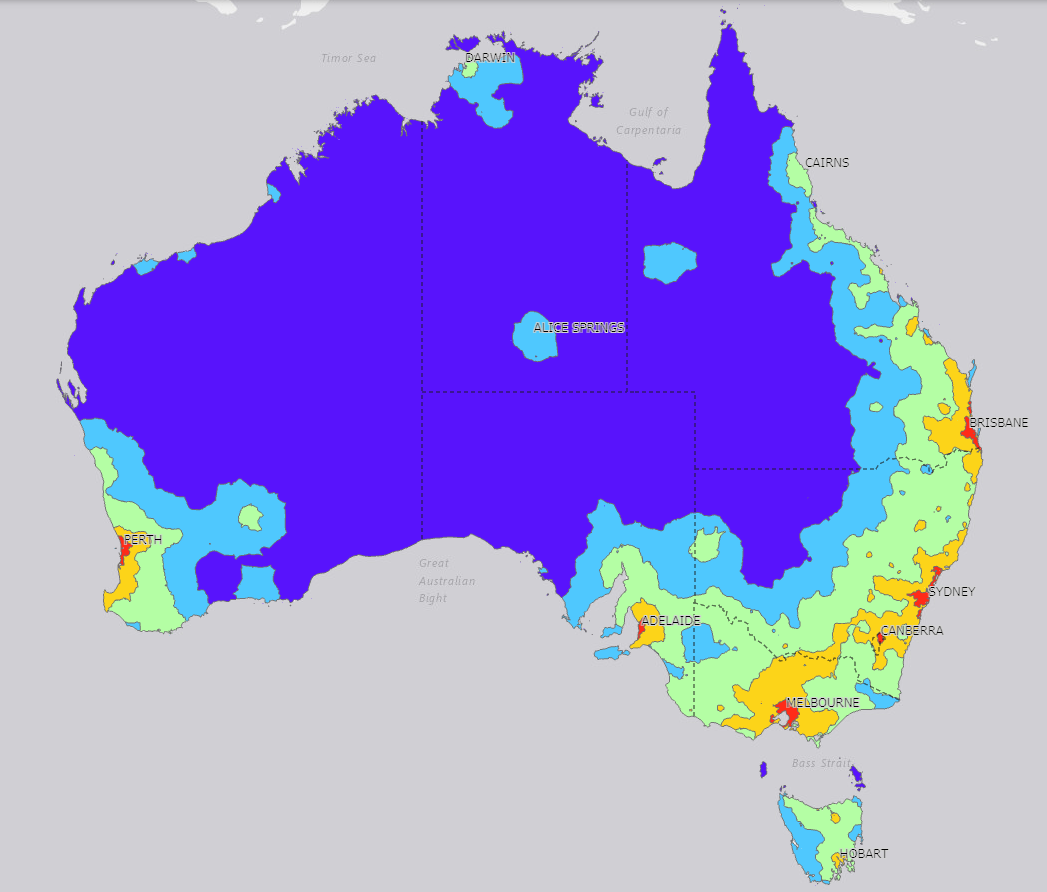


RA5 – Very Remote


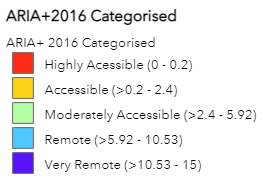


RA1 – Major cities

RA2 – Inner Regional

RA3 – Outer Regional

RA4 – Remote

RA5 – Very Remote

Available at:
<https://services.spatial.adelaide.edu.au/giscaportal/apps/webappviewer/index.html?id=417801ba9b844792af44ea4f766a3e30>

**ARIA+2016 Remoteness areas
of Australia**

**Figure S1: Map of the Australian Statistical Geography Standard (ASGS) Accessibility Remoteness Index of Australia (ARIA) 2016 Remoteness Areas Australia^26^.**

*ASGS ARIA+ 2016 defines 5 geographical categories or remoteness areas (RAs) determined by road distance from the closest urban centre^24,25^.
