# Supplementary figures and images for: Antenatal ultrasound needs-analysis survey of Australian rural/remote healthcare clinicians: recommendations for improved service quality and access
Source: BMC Public Health. 2023 Nov 17;23:2268. doi: 10.1186/s12889-023-17106-4 (PMC10655468; doi:10.1186/s12889-023-17106-4)

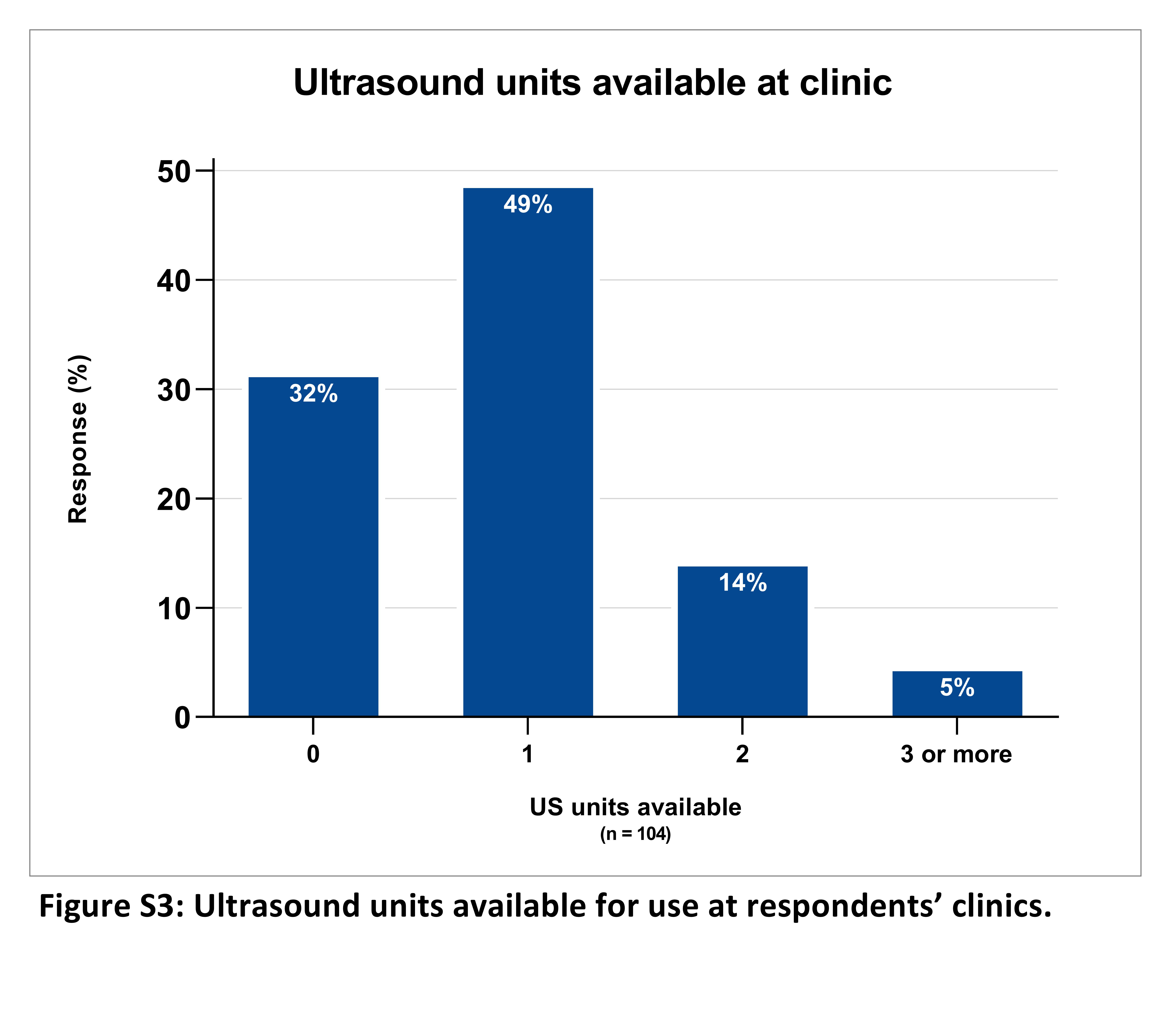

Supplement: Supplementary file 3 — Additional file 3: Figure S3. Ultrasound units available for use at respondents' clinics. [file 12889_2023_17106_MOESM3_ESM.jpg]

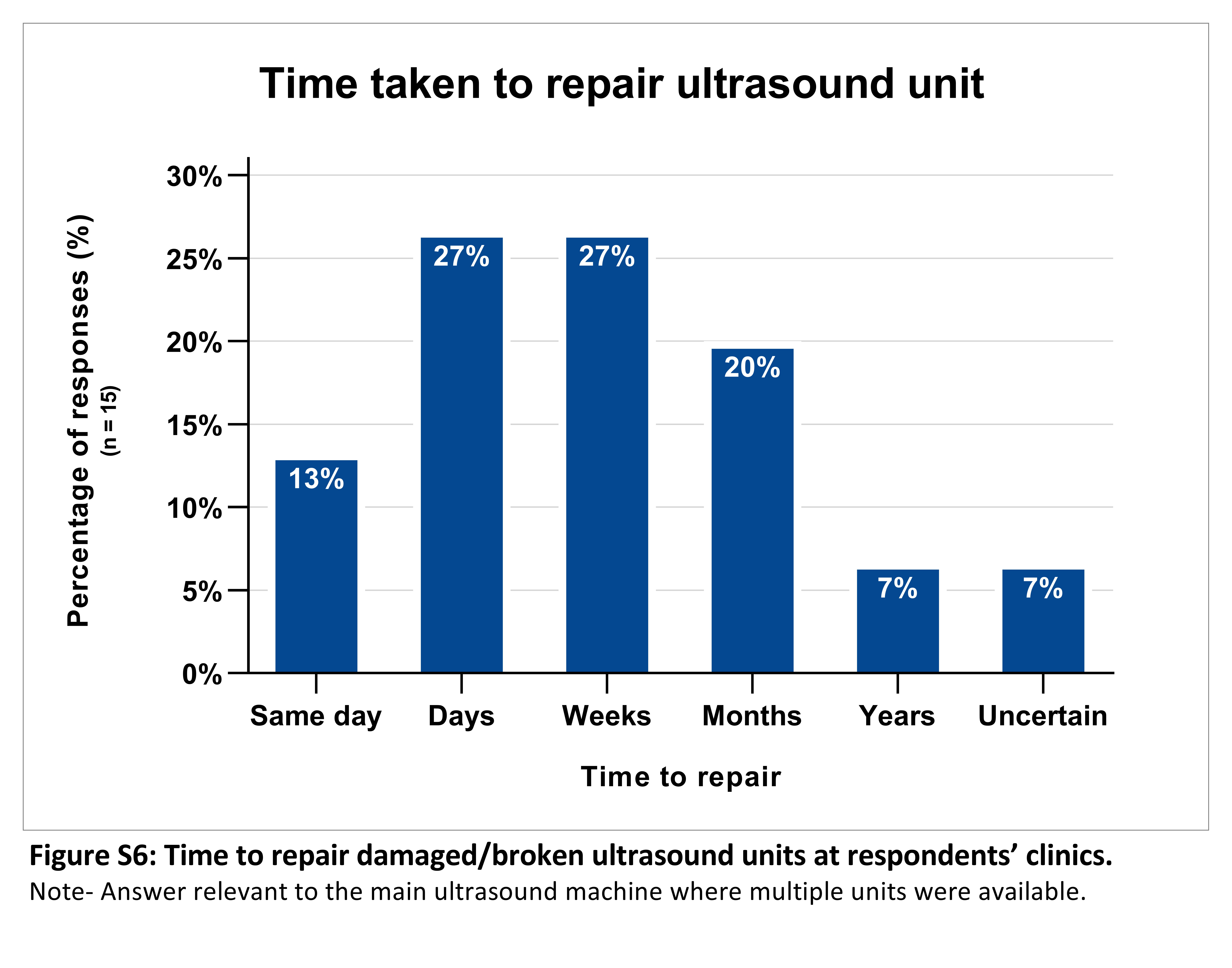

Supplement: Supplementary file 6 — Additional file 6: Figure S6. Time to repair damaged/broken ultrasound units at respondents' clinics. Note- Answer relevant to the main ultrasound machine where multiple units were available. [file 12889_2023_17106_MOESM6_ESM.jpg]

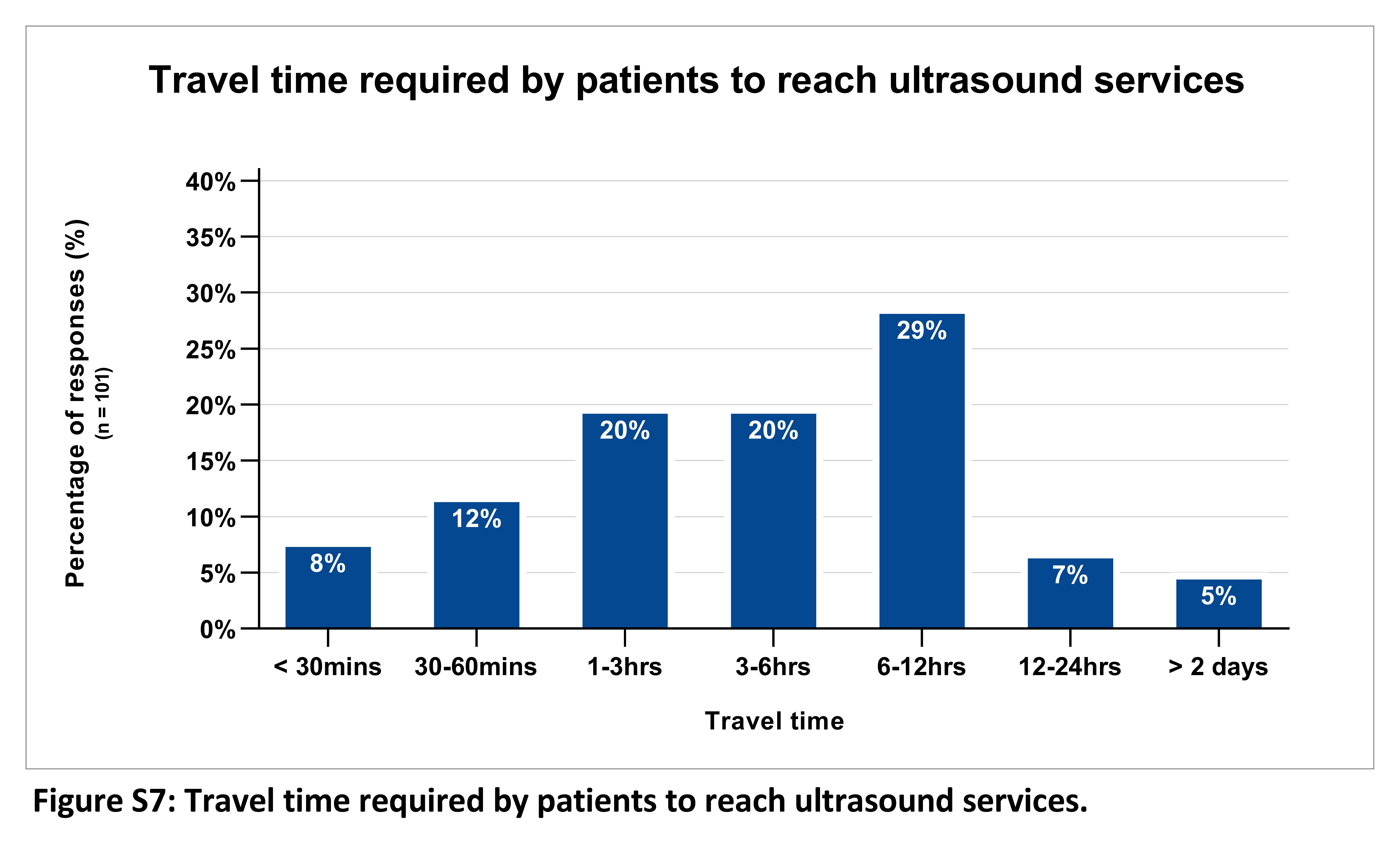

Supplement: Supplementary file 7 — Additional file 7: Figure S7. Travel time required by patients to reach ultrasound services. [file 12889_2023_17106_MOESM7_ESM.jpg]

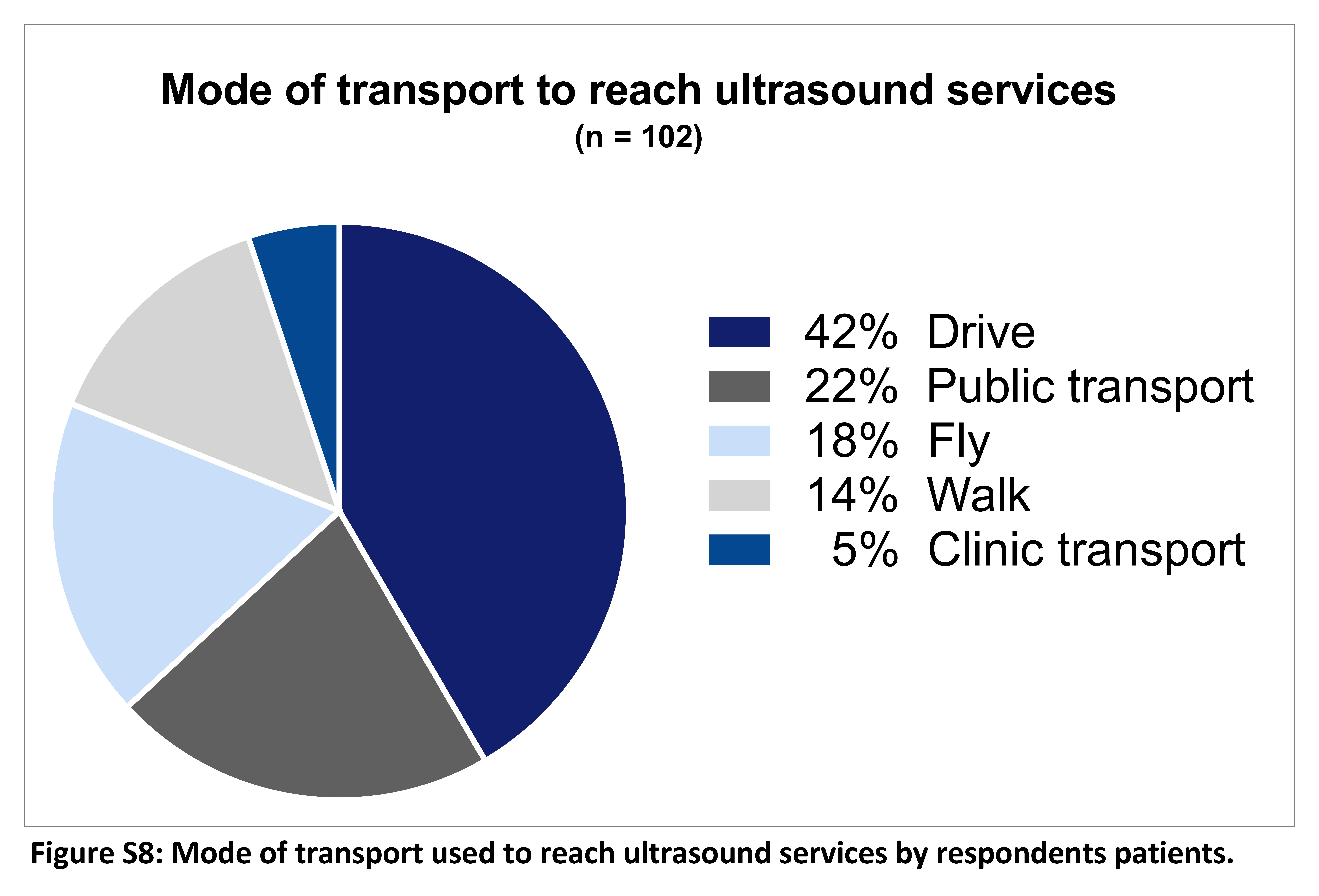

Supplement: Supplementary file 8 — Additional file 8: Figure S8. Mode of transport used to reached ultraound services by respondents patients. [file 12889_2023_17106_MOESM8_ESM.jpg]

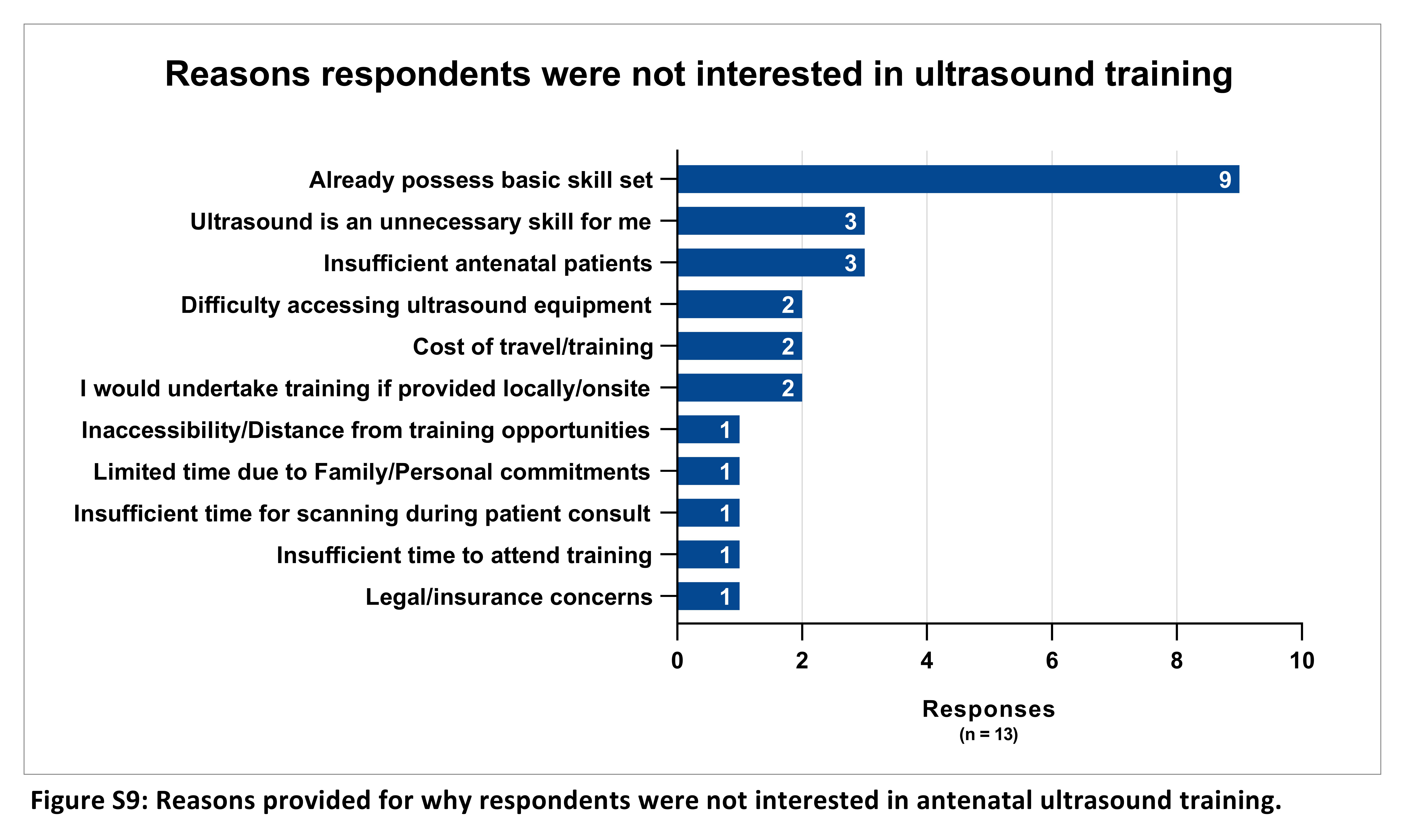

Supplement: Supplementary file 9 — Additional file 9: Figure S9. Reasons provided for why respondents were not interested in antenatal ultrasound training. [file 12889_2023_17106_MOESM9_ESM.jpg]
