# Supplementary material for: Antenatal ultrasound needs-analysis survey of Australian rural/remote healthcare clinicians: recommendations for improved service quality and access
Source: BMC Public Health. 2023 Nov 17;23:2268. doi: 10.1186/s12889-023-17106-4 (PMC10655468; doi:10.1186/s12889-023-17106-4)
Supplement: Supplementary file 11 — Additional file 11: Table S1. Perinatal, stillbirth and neonatal death rates per 1,000 births by Indigenous status and remoteness area (2020) [4]. Table S2. Maternal mortality ratio per 100,000 women giving birth by Indigenous status and remoteness area (2012-2020) [5]. [file 12889_2023_17106_MOESM11_ESM.docx]

**Table S1: Perinatal, stillbirth and neonatal death rates per 1,000 births by Indigenous status and remoteness area (2020)^4^.**

|  | **Indigenous status** | | **Remoteness area** | | | | |
| --- | --- | --- | --- | --- | --- | --- | --- |
|  | **Non-Indigenous** | **Aboriginal & Torres Strait Islander** | **Major cities**  **RA1** | **Inner regional**  **RA2** | **Outer regional**  **RA3** | **Remote**  **RA4** | **Very remote**  **RA5** |
| ***^a^Perinatal death** | 9.8 | 17.0 | 9.6 | 10.5 | 11.5 | 13.6 | 15.3 |
| ***^b^Stillbirths** | 7.4 | 11.9 | 7.3 | 8.0 | 8.3 | 9 | 11.1 |
| ***^c^Neonatal deaths** | 2.3 | 5.2 | 2.3 | 2.5 | 3.2 | 4.7 | 4.2 |

* The rate is the number of deaths per 1,000 births. Stillbirth and perinatal death rates were calculated using total births (live births and stillbirths). Neonatal death rates were calculated using live births.

Data extracted from- Australian Institute of Health Welfare. Australia's mothers and babies. Canberra: AIHW; 2022, Data tables: National Perinatal Mortality Data Collection annual update 2020, Available from: <https://www.aihw.gov.au/reports/mothers-babies/australias-mothers-babies/data>

**Table S2: Maternal mortality ratio per 100,000 women giving birth by Indigenous status and remoteness area (2012-2020)^5^.**

|  | **Indigenous status** | | **Remoteness area** | | | |
| --- | --- | --- | --- | --- | --- | --- |
|  | **Non-Indigenous** | **Aboriginal & Torres Strait Islander** | **Major cities**  **RA1** | **Inner regional**  **RA2** | **Outer regional**  **RA3** | **Remote & Very remote**  **RA4-5** |
| ****^d^Maternal mortality ratio**  **(MMR)** | 5.3 | 16.4 | 5.3 | 8.6 | 7.1 | 13.3 |

** MMR- Includes direct deaths, indirect deaths and not classified deaths, per 100,000 women giving birth between 2012 to 2020.

Data extracted from- Australian Institute of Health Welfare. Maternal deaths. Canberra: AIHW; 2022. Data tables: National Maternal Mortality Data Collection annual update 2020. Available from: <https://www.aihw.gov.au/reports/mothers-babies/australias-mothers-babies/data>

^a^ Perinatal death: stillbirth or neonatal death of a baby from 20 or more completed weeks of gestation to 28 days following birth or of 400 grams or more birthweight.

^b^ Stillbirth: a fetal death prior to birth of a baby of 20 or more completed weeks of gestation or of 400 grams or more birthweight.

^c^ Neonatal death: the death of a live born baby of 20 or more completed weeks of gestation or of 400 grams or more birthweight within 28 days of birth.

<https://www.aihw.gov.au/reports/mothers-babies/australias-mothers-babies/contents/technical-notes/definitions>

^d^ Definitions of Maternal mortality rates (Direct, indirect, not classified) available at: <https://www.aihw.gov.au/reports/mothers-babies/maternal-deaths-in-australia/contents/definitions-of-maternal-deaths>
