# Supplementary material for: Antenatal ultrasound needs-analysis survey of Australian rural/remote healthcare clinicians: recommendations for improved service quality and access
Source: BMC Public Health. 2023 Nov 17;23:2268. doi: 10.1186/s12889-023-17106-4 (PMC10655468; doi:10.1186/s12889-023-17106-4)
Supplement: Supplementary file 12 — Additional file 12: Table S3. Schedule of recommended antenatal ultrasound, components of screening and pregnancy complications. [file 12889_2023_17106_MOESM12_ESM.docx]

| **Schedule of formal antenatal ultrasound recommended in a low-risk pregnancy^1^** | |
| --- | --- |
| **First trimester** | For women uncertain of their date of conception, an 8-14 week ultrasound may be offered to determine gestational age and to perform or accurately schedule fetal anomaly screening (nuchal translucency ultrasound- most accurate at 11-13 weeks)^a^. |
| **Second trimester** | An 18 to 20 week ultrasound (morphology scan) should be offered to assess fetal movement, development and anatomy (structural anomalies). |
| **Third trimester** | For suspected non-cephalic presentation after 36 weeks where abdominal palpation is inconclusive, an ultrasound assessment should be used to confirm fetal position and assess for potential life-threatening delivery complications such as nuchal cord and placenta previa. |
| **Components of antenatal ultrasound screening^1,38^** | |
| - Gestational age/Estimated due date/Date of conception (can perform from 6 weeks, more accurate before 12 weeks/first trimester) - Fetal size and Fetal growth - Cardiac activity - Fetal number (multiple pregnancy) - Chorionicity if multiple pregnancy - Placental appearance and location (more informative after 18 weeks/second trimester) - Fetal well-being/Viability - Fetal anomalies- Presence of normal head, neck, face, spine, chest, heart, abdomen, abdominal wall, and extremities (more informative after 18 weeks) | |
| **Pregnancy complications^1,4^** | |
| - Ectopic pregnancy - Multiple pregnancies - Inter Uterine Growth Restriction (IUGR) - Congenital anomalies - Pre-term labour - Post-term pregnancy - Pre-eclampsia/Eclampsia Nuchal cord - Malpresentation - Polyhydramnios - Placenta praevia - Placenta abruption - Gestational diabetes | |
| ^a^ “The timeframe for ultrasound assessment of gestational age overlaps with that for assessment of nuchal translucency thickness as part of testing for fetal chromosomal anomalies (11 weeks to 13 weeks 6 days), which may enable some women to have both tests in a single scan. This should only occur if women have been provided with an explanation of the purpose and implications of the tests and have given their informed consent to both tests.”  <https://www.health.gov.au/sites/default/files/pregnancy-care-guidelines_0.pdf> | |

**Table S3: Schedule of recommended antenatal ultrasound, components of screening and pregnancy complications.**
