# Supplementary material for: Antenatal ultrasound needs-analysis survey of Australian rural/remote healthcare clinicians: recommendations for improved service quality and access
Source: BMC Public Health. 2023 Nov 17;23:2268. doi: 10.1186/s12889-023-17106-4 (PMC10655468; doi:10.1186/s12889-023-17106-4)
Supplement: Supplementary file 13 — Additional file 13: Table S4. Characteristics of survey respondents. [file 12889_2023_17106_MOESM13_ESM.docx]

**Table S4: Characteristics of survey respondents.**

| Demographic | Number (n/114) | Per cent  (%) |
| --- | --- | --- |
| Gender | | |
| Female | 98 | 86% |
| Male | 16 | 14% |
| Years of clinical experience |  |  |
| 0-5 | 12 | 11% |
| 6-10 | 24 | 21% |
| 11-20 | 31 | 27% |
| 21-30 | 18 | 16% |
| 31-40 | 22 | 19% |
| 40+ | 7 | 6% |
| Employment role (Note: 25 respondents reported dual/multiple roles) | | |
| Clinical Manager | 10 | 7% |
| General Practitioner | 40 | 28% |
| Registered Midwife | 52 | 36% |
| Nurse | 25 | 18% |
| OBGYN | 6 | 4% |
| Sonographer | 1 | 1% |
| ATSIW- Aboriginal or Torres Strait Islander Worker | 6 | 4% |
| CHW- Community Healthcare Worker | 2 | 1% |
| Remoteness area worked (*ASGS ARIA 2016+) | | |
| RA2 – Inner regional | 6 | 5.3% |
| RA3 – Outer regional | 21 | 18.4% |
| RA4 – Remote | 26 | 22.8% |
| RA5 – Very remote | 61 | 53.5% |
| *ASGS ARIA+ 2016- The Australian Statistical Geography Standard (ASGS) Accessibility Remoteness Index of Australia (ARIA) defines 5 geographical categories or remoteness areas (RAs) determined by road distance from the closest urban centre^24,25^. | | |
