# Supplementary material for: Antenatal ultrasound needs-analysis survey of Australian rural/remote healthcare clinicians: recommendations for improved service quality and access
Source: BMC Public Health. 2023 Nov 17;23:2268. doi: 10.1186/s12889-023-17106-4 (PMC10655468; doi:10.1186/s12889-023-17106-4)
Supplement: Supplementary file 14 — Additional file 14: Table S5. Limitations to existing travel arrangements at respondents’ clinics. [file 12889_2023_17106_MOESM14_ESM.docx]

| **Table S5: Limitations to existing travel arrangements at respondents’ clinics.** |
| --- |
| ▪ Travel assistance:  - Only available to complex pregnancy, breastfeeding mothers, Aboriginal women.  - Capped prices: costs not fully covered, cost of petrol only.  - Funding only for cheapest travel option (and maybe inappropriate for heavily pregnant or unwell patients).  - Funding only available for travel to the closest radiology service.  - Funding only available for travel to Patient Assistance Transport Scheme determined radiology service.  - Strict eligibility criteria as defined by Patient Assistance Transport Scheme policy.  - Only travel for specific scans covered unless ordered by a specialist.  - Patients having missed previous appointments are ineligible for future travel assistance. |
| ▪ Accompanying person/Escort:  - No accompanying person for scans and limited escorts for births.  - Children unable to travel with patients (mothers have no safe childcare options and are reluctant to leave other children at home).  - Accompanying person only for first pregnancy, underage patients or complex patients. |
| ▪ Public transport:  - No public transport/Limited public transport (no available tickets, limited days operation per week, lack of daily transport options- schedule limits access to appointment times and length).  - Disruptions to bus schedule (unreliable).  - Distance to travel to reach public transport. |
| ▪ Flights:  - Missed flights lead to 10 days wait to rebook.  - Full plane needed for charter. |
| ▪ Limited staff and funding to run clinic provided transport. |
| ▪ Limited accommodation options when overnight stay required. |
| ▪ Weather prohibiting travel. |
| ▪ Cost prohibiting travel. |
