# Supplementary material for: Antenatal ultrasound needs-analysis survey of Australian rural/remote healthcare clinicians: recommendations for improved service quality and access
Source: BMC Public Health. 2023 Nov 17;23:2268. doi: 10.1186/s12889-023-17106-4 (PMC10655468; doi:10.1186/s12889-023-17106-4)
Supplement: Supplementary file 15 — Additional file 15: Table S6. Impacts of ultrasound on patient lifestyle reported/perceived by respondents/clinicians. [file 12889_2023_17106_MOESM15_ESM.docx]

| **Theme** | **Frequency (n/N)** | **Per cent  (%)** |
| --- | --- | --- |
| **Yes - Positive impact** | **71/100** | **70%** |
| **Correlation between visualising the fetus and behaviour/lifestyle**: confirmation of the pregnancy and personification of the fetus leading to an increased sense of responsibility and improved motivation, visualisation of improvement with consecutive monitoring confirming positive lifestyle behaviours. | 34/71 | 48% |
| **Increased engagement and bonding**: Enhanced maternal engagement with the pregnancy, maternal and paternal bonding. | 16/71 | 23% |
| **Patient education/opportunity to discuss lifestyle management and impact on lifestyle decisions.** | 14/71 | 20% |
| **Reduction/cessation of harmful behaviours**: earlier reduction/cessation of substance use (alcohol, smoking). | 10/71 | 14% |
| **Identification of pregnancy risks/complications/comorbidities**. | 5/71 | 7% |
| **Reduced stress and anxiety**. | 4/71 | 6% |
| **Improved antenatal care compliance**. | 2/71 | 3% |
| **Yes - Negative Impact** | **1/100** | **1%** |
| Reassurance provided by a ‘normal’ ultrasound reinforce existing harmful behaviours  Stress of identified complications can lead to poor lifestyle choices | 1/71 | 1% |
| **No impact** | **20/100** | **20%** |
| Perceived lack of motivation | 6/20 | 30% |
| Other influences being more significant to lifestyle | 6/20 | 30% |
| Inability to change current lifestyle | 3/20 | 15% |
| Sociocultural factors | 3/20 | 15% |
| Lack of evidence/not convinced of the association | 3/20 | 15% |
| Problems being identified too late in pregnancy | 1/20 | 5% |
| *Note: 9/100 respondents selected uncertain* |  |  |

**Table S6: Impacts of ultrasound on patient lifestyle reported/perceived by respondents/clinicians.**
