# Supplementary material for: Antenatal ultrasound needs-analysis survey of Australian rural/remote healthcare clinicians: recommendations for improved service quality and access
Source: BMC Public Health. 2023 Nov 17;23:2268. doi: 10.1186/s12889-023-17106-4 (PMC10655468; doi:10.1186/s12889-023-17106-4)
Supplement: Supplementary file 16 — Additional file 16: Table S7. Sample responses of ultrasound impact on patient lifestyle choices/behaviour as reported/perceived by respondents/clinicians. [file 12889_2023_17106_MOESM16_ESM.docx]

**Table S7: Sample responses of ultrasound impact on patient lifestyle choices/behaviour as reported/perceived by respondents/clinicians.**

| **Theme** | **Sample survey responses** |
| --- | --- |
| **Positive impact** | |
| **Visualisation of the fetus** | *“Seeing their unborn baby moving conveys the sense of reality, forcing most to reconsider all aspects of their diets and lifestyle choices...”*  *“When they can see the baby moving and kicking and sucking its hands, it does aid in assisting with changing to healthier lifestyle choices.”*  *“Often when they see their baby on the scan, they get more protective & start making healthier lifestyle choices.”*  *“Yes, it may make a difference on the first dating scan as they have a visual which may make them change lifestyle factors.”* |
| **Increased engagement and bonding** | *“Seems to help the reality and bonding with baby.”*  *“Helps them [patients] to engage with the baby earlier, helps to visualise what is going on so motivation can be better.”*  *“Early bonding - for fathers also.”*  *“Seeing the baby makes it more real, they feel more responsibility and connection with the baby.”* |
| **Patient education/opportunity to discuss lifestyle management and impact on lifestyle decisions** | *“I discuss and educate at the same time and the women feel a stronger connection to the baby.”*  *“When growth is monitored, for example invites the conversation about lifestyle management.”*  *“It has them even more involved in the health aspects of pregnancy, affords more opportunity to discuss these issues.”*  *“If we can show development and explain importance visually it can improve engagement.”* |
| **Reduction / Cessation of harmful behaviours** | *“In early pregnancy it can motivate cessation of substance use as it can make the pregnancy seem more real.”*  *“Helps them to stop smoking and drinking by visualising the baby.”*  *“I have experiences in IUGR* [Intrauterine Growth Restriction] *babies whose mothers reduced smoking.”*  *“One patient had no interest in her baby until she saw it was a girl and decided to stop drinking. Majority of patients already want to do what’s best for bub.”* |
| **Identification of pregnancy risks /complications/ comorbidities** | *“Especially in GDM* [Gestational Diabetes Mellitus], *multiple pregnancy or complications.”*  *“Confirming pregnancy or reassurance following a bleed is often a motivator for behaviour change.”*  *“If the baby's wellbeing was a concern, the mother would be encouraged to adapt her lifestyle choices as needed.”*  *“If they know there are issues they can make better choices and can see the result of better choices.”* |
| **Reduced stress and anxiety** | *“Relieves stress and anxiety about pregnancy and their baby.”*  *“Less stress of travel, more convenient.”*  *“Especially in later pregnancy when women begin to get concerned re: size of baby.”*  *“They find out if their babies are okay and this helps their mental health.”* |
| **No impact** | |
| **Perceived lack of motivation** | *“Intention to improve lifestyle, but often not followed through once l leaving the consult.”*  *“I don't think it worries some people.”*  *“No, because it is not perceived as important unless there is a problem.”* |
| **Other influences being more significant to lifestyle** | *“There are other less invasive measures of fetal wellbeing and trusted known and continuity of carer much more important.”*  *“Those decisions more based on pregnancy than ultrasound.”*  *“Comprehensive education and counselling is more effective.”*  *“Other mindsets and influences more significant.”* |
| **Inability to change current lifestyle** | *“Some … women will not change lifestyle no matter how much health education / scans are offered.”*  *“Most have had previous pregnancies and not changed their behaviours/know someone who have had healthy babies regardless of their behaviours.”*  *“Women already are aware they are pregnant and still have not made any lifestyle changes so USS will not make any difference.”* |
| **Sociocultural factors** | “*Choices determined by other social factors.”*  *“So far it seem like no. Possibly due to cultural reasons.”* |
| **Lack of evidence/not convinced of the association** | *“There are no studies I’m aware of demonstrating that it does, in personal experience, no.”*  *“Not convinced of association between lifestyle and outcome.”*  *“Some women don’t associate their behaviours with poor outcomes.”* |
| **Problems being identified too late in pregnancy** | *“It has the potential but often issues around reduced size associated with maternal smoking can only be fully realised too late in the pregnancy (often in the 3rd trimester).”* |
| **Negative impact** | |
| **Reassurance of normal scan / Stress of an abnormal scan** | *“Sometimes it is reassuring so they can continue to eat poorly, not bond with the baby. Other times, women find that no matter what they do, the baby continues to suffer low weight gain - that the environment that they have provided isn't 'good enough'. Also an issue if a woman is stressed because something 'high risk' has been found - so poor lifestyle choices are more because of stress.”* |
| **Uncertain of impact** | |
| **Uncertain** | *“I'm not sure as it's very difficult to change lifestyle practices when there is little choice or support.”*  *“Maybe. Remote antenatal care is a tough gig.”* |
