# Supplementary material for: Antenatal ultrasound needs-analysis survey of Australian rural/remote healthcare clinicians: recommendations for improved service quality and access
Source: BMC Public Health. 2023 Nov 17;23:2268. doi: 10.1186/s12889-023-17106-4 (PMC10655468; doi:10.1186/s12889-023-17106-4)
Supplement: Supplementary file 17 — Additional file 17: Table S8. Additional comments from survey participants regarding the provision of ultrasound services to pregnant women in rural Australia. [file 12889_2023_17106_MOESM17_ESM.docx]

| **Table S8: Additional comments from survey participants regarding the provision of ultrasound services to pregnant women in rural Australia.** |
| --- |
| **Concluding additional comments (optional) by survey participants** |
| *“Reinforce the comments made: need recognition of this skill, should be remunerated significantly given that this service is an important adjunct, sometimes lifesaving and reduces burden on state-wide services, e.g. flying cases off-island for management, PATS* [Patient Assisted Travel Scheme] *for off-island scans. Should also be supported with ongoing training and Locum cover in order to maintain skill. Also strongly think rural women are disadvantaged in paying heavily for scans in pregnancy at private imaging services. It is like a monopoly - the women have no choice. This is wrong and should be subsidised I believe.”* |
| *“I think that ultrasound is an essential tool, as an extension of clinical examination. It is becoming more affordable and portable and should be taught to all rural GPs* [General Practitioners]*. Medicare rebates would be helpful to encourage practices to start using it if they are not already.”* |
| *“Sad to see it becoming routine assessment tool, this is not supported by evidence and risks deskilling of care providers, also not well explored any risks of routine serial ultrasound.”* |
| *“Obviously more difficult than in city regions often dealing with obstetric emergencies with limited skills.”* |
| *“I think all remote midwives should be trained and have access to ultrasound for visiting clinics to increase the likelihood of indigenous women seeking early antenatal care, rather than avoiding it so they don’t have to go to town and leave behind their other children.”* |
| *“Benefits: Cost saving, early parental bonding supports lifestyle changes need: QA* [Quality Assurance] *programs, policy/procedure of use.”* |
| *“Having it more accessible.”* |
| *“I think providing ultrasound services to women in rural/remote Australia is a fantastic idea it provides the women with options, decreases the stress of them having to leave their community and family.”* |
| *“For women with well routine low risk pregnancies ultrasound services in this location are excellent There are issues for high risk women with flights and need for frequent ultrasound services including cost of travel accommodation.”* |
| *“Barrier to accessing services is a huge issue. Distance and travel time etc…”* |
| *“Longstanding shortage of services significantly affects quality of antenatal care.”* |
| *“I absolutely think that ultrasound services should be available & more accessible to pregnant women in rural/remote Australia because if there are any issues identified our ladies have so much further to travel to access services that are available to the women who live in cities & are much closer. Often this presents major problems with Mum's who already have little ones at home & become very stressed & upset about having to leave their families to travel so far away. If these issues are picked up earlier for the ladies it isn't so traumatic for them, & if they don't have to travel so far for an ultrasound & pay so much money there will be a lot less stress experienced. Some ladies won't/can't have the ultrasounds if they have to pay because they just don't have the money.”* |
| *“It is available to all metro based women - it should be for rural and remote women too.”* |
| *“The lack of funding for the time spent scanning (i.e. appropriate items to be able to bill) and the cost of the machine are the biggest barriers to providing ultrasound.”* |
| *“Ultrasounds are over utilised in Maternity services and yet we know very little about the over-all risks to fetal (and maternal) wellbeing. Bedside scanning is now a routine while clinical assessment skills are being lost by Obstetrical teams - Let's begin to refocus on clinical skills that lead to high calibre assessments. This could include continuity of care models of service; more support in developing abdominal palpation acuity where medical students are coached in basic skills of fetal wellbeing. positioning and decent - which goes hand in hand with the art of Vaginal examination (which is respectful and discerning) i.e. status of the cervix in relation to the presenting part; presentation, position, is the presenting part flexed or deflexed, etc, etc, etc - the outcome being a collective of information that can be used to provide appropriate care to a woman and her unborn - rather than the immediate reach for an ultrasound machine.”* |
| *“Get indemnity insurers on board.”* |
| *“We do use ultrasound in our clinic and I have been asked to use it when the Doctor is not in attendance and shown him the screen on facetime so that he could diagnose. To have some formal training would be extremely helpful.”* |
| *“Would be great to offer uss* [Ultrasound scans] *to all ladies at AN visits if they ask, even as a reassurance.”* |
| *“I think people with knowledge of pregnancy are best to do uss for ante nates.”* |
| *“I think in general the uptake is very good despite remote location in Australia. It is well accepted by our women as very important. Most women who may have limited antenatal care, will always present when due for a routine scan. Sometimes there are limitations of access to our private imaging service due to the fact the sonographer is male. In saying this, there is access to chaperones.”* |
| *“We have a long delay in accessing routine uss at local hospital - up to 6 weeks for routine appointments; antenatal patients are prioritised but bookings are hard to come by and it is frustrating when people refuse to travel.”* |
| *“Almost everywhere has a laptop ultrasound machine these days and it is very important to be able to use it as it gives reassurance to staff and patients alike and saves patients, clinics, and health service money in long run. In our case it often negates he need for RFDS* [Royal Flying Doctors Service] *fly out.”* |
| *“Rural and remote pregnant women are disadvantaged when trying to access ultrasound services. Most women here don't know their LMP* [Last Menstrual Period]*, and if they have to be financially supported to obtain a dating ultrasound, which enables accurate birth planning and pregnancy surveillance throughout the pregnancy, then they will not be funded to access a NTS* [Nuchal Translucency Scan] *unless they are high risk. This takes choice out of the equation for many women. Additionally they have to go to the city on their own without an escort which is often frightening and problematic. Other issues are leaving their other children behind to travel, when sometimes the children’s safety is of concern. This issue does prevent some women from accessing their scans. Once I do access my training, I am looking forward to being able to perform a dating scans, to enable all my women to have funded trips for NTS if they choose, even low risk women, and growth surveillance to prevent women leaving their other children so often, even though I can't assist with the escort issue. Thankyou.”* |
| *“This is essential training for midwives and GPs who work in remote locations. Being able to provide an early and often opportunistic scan is able to make such a difference to care.”* |
| *“The shortage of sonographers in rural regions is a huge factor is provision of services, more needs to be done to attract young sonographers at the start/middle of their careers.”* |
| *“As a Remote Area Nurse who is already a licenced X-ray operator, I have seen the benefits this has bought to the communities by having immediate diagnosis, immediate definitive care, immediate known need for referral to appropriate clinic, decrease time off community, decreased members go tom town and "getting lost". For all these similar types of reasons I would see for having ultrasound equipment and skills onsite.”* |
| *“Needs more support, better funding and perhaps MBS* [Medicare Benefits Schedule] *item funding.”* |
| *“It’s very hard work getting women into town, getting children looked after, making a trip into town be about the ultrasound not, a bonus holiday. Indigenous women, hate the waiting in the strange hospital environments, are often found outside and can be assumed missing or not attending when called.”* |
| *“I have worked in the rural and remote setting for 10 years and it is a persistent problem with accessing appointments for scans but we now have a visiting ultrasound service once a month to 2 rural areas which has been a great benefit for the communities.”* |
| *“I believe Diagnostic ultrasound skills would highly benefit clinic assessment for many presentations and assist in decision to transfer for many remote area sites.”* |
| *“Having access to a portable machine would make outreach antenatal care a whole lot more accessible to remote Aboriginal communities, and is an important to bring antenatal care to the women rather than have to travel them long distances in.”* |
| *“I feel that all RM in outreach/community clinic need this skill. I am working quite remote and would LOVE to be able to perform ultrasound.”* |
| *“I think it has been great + it's used in another community by different provider that I visit as part of antenatal care. We can both discuss the cases/results. It's given confirmation to clinical risk factors and have been able to act more quickly.”* |
| *“I would love to have training in ultrasound so that I can practice in my clients and my community would be benefitted.”* |
| *“More opportunities for funded training for midwives would increase accessibility to ultrasound services for women in their own community.”* |
