# Supplementary material for: Antenatal ultrasound needs-analysis survey of Australian rural/remote healthcare clinicians: recommendations for improved service quality and access
Source: BMC Public Health. 2023 Nov 17;23:2268. doi: 10.1186/s12889-023-17106-4 (PMC10655468; doi:10.1186/s12889-023-17106-4)
Supplement: Supplementary file 18 — Additional file 18: Table S9. Recommendations from national survey. [file 12889_2023_17106_MOESM18_ESM.docx]

| **Table S9: Recommendations from national survey.** |
| --- |
| 1. **Upskilling rural healthcare clinicians** |
| 1.1. Promoting the existence and availability of PoCUS courses and accreditation pathways could increase the number of rural clinicians pursuing training to advance their clinical skills. |
| 1.2. Broadening the scope of practice for nurses and midwives (along with GPs/doctors) to include basic antenatal ultrasound services, with appropriate training and clear practice definitions, would increase workforce capacity in these under-resourced areas. Expanding roles and scope of practice can also help to empower health professionals leading to greater job satisfaction and rural workforce retention. |
| 1.3. Subsidising ultrasound training schemes for rural clinicians to cover training and travel costs could help to incentivise clinicians towards upskilling. |
| 1.4. Providing local/rural-based training programs would overcome the need for frontline rural healthcare clinicians to leave work and family commitments, and for locum staff replacements (often at significant additional expense) to cover their absences while travelling to urban training centres. |
| 1.5. Increasing access to ASUM’s PoCUS accreditation pathways formally certifying clinicians’ skills would assist in consolidating clinical competency and provide clinicians with additional distance support. |
| 1.6. Establishing and simplifying access to financial incentives with Medicare for remote PoCUS use could encourage clinicians to purchase equipment, undertake training and take time to scan patients during consults. |
| 1.7. Telehealth and Teleultrasound may offer a solution to reduce travel requirements for rural clinicians interested in training and formal certification and warrants further investigation. |
| 1.8. Education campaigns on the advantages of Telehealth/Teleultrasound for education, post-training support, clinical supervision and access to specialists/experts could assist uptake of the technology by rural clinicians. |
| 1.9. Government support to assist rural clinics in implementing telehealth systems, and improving telecommunication infrastructure is indicated. |
| 1.10. Access to centralised medical repositories like the government’s ‘[My Health Record](https://www.myhealthrecord.gov.au/)’. |
| 1. **Ultrasound equipment** |
| 2.1. In rural clinics where there are staff trained in the use of PoCUS but no available equipment, a relatively small initial expenditure supplying equipment could provide substantial benefit. |
| 2.2. The provision of portable ultrasound units would allow clinicians to travel with the equipment and provide opportunistic scanning at community/home/outreach visits. |
| 2.3. Where staff have not received appropriate training, the provision of combined equipment and subsidised training is warranted. |
| 2.4. Replacement and renewal of ultrasound equipment should be carried out every 5-10 years, and formal replacement programs may be warranted. |
| 1. **Patient access** |
| 3.1. Existing patient transport schemes would benefit from review to: increase subsidies to cover more of the travel costs; make the schemes more visible to patients and staff; simplify application processes; broaden eligibility criteria making them accessible to more rural women; and provide allowances for an escort (for schemes that limit this option). |
| 3.2. Review of scan costs (patients’ out-of-pocket costs) and Medicare rebates (both formal and PoCUS) for rural patients is advisable. |
| 3.3. Outreach/home/community visits by trained clinicians with portable ultrasound equipment could greatly benefit remote and Aboriginal populations. |
| 3.4. Rural communities may also benefit from targeted, culturally sensitive education campaigns to increase patient awareness of the benefits of antenatal care and ultrasound early in pregnancy. |
| 3.5. Greater focus on providing culturally sensitive care to women in rural communities is indicated, ideally through increasing the number of Aboriginal healthcare workers in these regions and targeting these workers for PoCUS training. |
| 3.6. Education initiatives teaching cultural sensitivity should be implemented for the existing and future rural workforce. |
