# Supplementary material for: Antenatal ultrasound needs-analysis survey of Australian rural/remote healthcare clinicians: recommendations for improved service quality and access
Source: BMC Public Health. 2023 Nov 17;23:2268. doi: 10.1186/s12889-023-17106-4 (PMC10655468; doi:10.1186/s12889-023-17106-4)
Supplement: Supplementary file 19 — Additional file 19. Needs Analysis Survey pro forma. [file 12889_2023_17106_MOESM19_ESM.pdf]

Needs Analysis Survey pro forma

University of South Australia

UniSA HEALTHY NEWBORN PROJECT National antenatal ultrasound needs analysis survey

Information on antenatal ultrasound (US) services in rural and remote Australian communities

Welcome and thank you for your participation. This survey is being conducted by**The Healthy Newborn Project** research team from the Division and School of Health Sciences at**the University of South Australia (UniSA)**, School of Health Sciences, to examine availability, accessibility and utilisation of antenatal ultrasound services and resources in regional, rural and remote areas of Australia.

Data collected through this survey will provide robust evidence to inform governments of future planning needs and solutions. Obtaining feedback from professionals in frontline rural and remote health care is vital to this process. Let your voice be heard.

The survey is estimated to take approximately**5-20 minutes** of your time and we greatly appreciate your effort in its completion. **If your clinic does not perform US (no US machine available or no trained staff) but provides antenatal care, please still complete the survey.** On completion of the survey, you will have the opportunity to enter the draw to receive a **$1000 prize/scholarship**. The scholarship can be used to attend a conference or course of your choice to benefit your clinic and assist with your Continuing Professional Development. This scholarship is to be redeemed for educational purposes only and is for participants in resource poor areas (rural and remote locations).

Confidentiality statement: **Only healthcare professionals are being surveyed**, no patients will be approached or questioned for this research. **Individual responses will remain confidential and will be analysed collectively** with other participant responses. The researcher will take every care to remove identifying material from the responses provided as early as possible and participants will not be identified in the reporting of the research. No information which could lead to the identification of any individual will be released, unless as required by law. However, the researcher cannot guarantee the confidentiality or anonymity of material transferred by email or the internet. Participation is voluntary and you are free to withdraw from the study at any point while completing the survey, without affecting your position now or in the future. However once submitted, we are unable to remove your response as it will be impossible to identify your individual data. It is not anticipated that there are any risks to participation in this study beyond those encountered during everyday life.

All information collected as part of the study will be retained for five years. Data will be stored securely onsite in electronic format on a password protected UniSA computer server. Data will be held on survey monkey platform until exported, then deleted immediately following collection and export. The data collected will be non-identifiable.

This study is funded by 'The Hospital Research Foundation'. This research is in line with the National Statement on Ethical Conduct in Human Research (2007) - Updated May 2015 (https://www.nhmrc.gov.au/guidelines-publications/e72). This project has been approved by the University of South Australia's Human Research Ethics Committee. If you have any ethical concerns about the project or questions about your rights as a participant, or should you or any third parties wish to lodge a complaint about either the study or the way it is being conducted, please contact the Executive Officer of this Committee – Ms Vicki Allen (tel: +61 8 8302 3118; email: humanethics@unisa.edu.au). If you have any other questions or concerns, please contact Amber Bidner at email: Amber.Bidner@unisa.edu.au.

* 1. What is your current employment position? (multiple options may be selected if applicable)

- ☐ Clinic Manager (CM)
- ☐ General Practitioner (GP)
- ☐ Registered Midwife (RM)
- ☐ Nurse
- ☐ Obstetrician/Gynaecologist (OB/GYN)
- ☐ Sonographer
- ☐ Aboriginal Health Worker
- ☐ Community Health Worker (CHW = any health care worker who works with pregnant women and is not a GP, RM, Nurse or specialist)
- ☐ Other (please specify) or any additional comments

* 2. What is the postcode of your clinic/s?

(Please note this information will be confidential and only used to generate an ARIA remoteness score for demographics)

* 3. How many years of clinical experience do you have?

* 4. What is your gender? (for workforce distribution analysis only)

- ◯ Male
- ◯ Female
- ◯ Non-binary
- ◯ Other
- ◯ Prefer not to say

University of South Australia

UniSA HEALTHY NEWBORN PROJECT National antenatal ultrasound needs analysis survey

AVAILABILITY OF ANTENATAL ULTRASOUND

Ultrasound use within your clinic

*** 5. How many hours per month on average (estimate) is antenatal care delivered within your clinic?**

*** 6. Is ultrasound used when antenatal care is delivered?**

- Yes
- No

Any additional comments (optional)

University of South Australia

UniSA HEALTHY NEWBORN PROJECT National antenatal ultrasound needs analysis survey

AVAILABILITY OF ANTENATAL ULTRASOUND

*** 7. Who performs the antenatal ultrasound scans when antenatal care is delivered? (select all applicable options)**

- ☐ Onsite General Practitioner (GP)
- ☐ Onsite Registered Midwife (RM)
- ☐ Onsite OBGYN Specialist
- ☐ Onsite nurse
- ☐ Onsite sonographer
- ☐ Visiting General Practitioner (GP)
- ☐ Visiting Registered Midwife (RM)
- ☐ Visiting OBGYN Specialist
- ☐ Visiting nurse
- ☐ Visiting sonographer
- ☐ Other (please specify)

*** 8. How many antenatal ultrasound scans are performed in your clinic per month on average (estimate)?**

*** 9. How many antenatal scans do low-risk pregnant women attending your clinic receive per pregnancy, on average?**

- ○ 0
- ○ 1
- ○ 2
- ○ 3
- ○ 4 or more

Any additional comments (optional)

*** 10. In which trimesters are antenatal scans usually performed for low risk uncomplicated pregnancies? (select all applicable options)**

	1 scan	2 scans	3 scans	4 or more scans
Trimester 1	○	○	○	○
Trimester 2	○	○	○	○
Trimester 3	○	○	○	○

*** 11. How do women react to the scan? (e.g. excited, makes the pregnancy real, anxious)**

*** 12. Which clinical indications lead to antenatal ultrasound in your clinic? (select one option for each indication/row listed below)**

	Common	Uncommon	Not Applicable
Estimated due date	○	○	○
Fetal growth	○	○	○
Identify multiple pregnancy	○	○	○
Placenta location	○	○	○
Positioning- Fetal lie/Presentation	○	○	○
Loss of fetal movement	○	○	○
Identification of fetal heartbeat	○	○	○
Amniotic fluid assessment	○	○	○
Fetal anomalies/defects	○	○	○
History of PV bleeding	○	○	○
Ectopic pregnancy	○	○	○
Maternal trauma/injury/illness	○	○	○

Any additional comments (optional)

*** 13. What barriers, if any, are faced when utilising antenatal ultrasound in your clinic? (select all applicable options)**

- [ ] None- There are no significant barriers
- [ ] Difficulty accessing the ultrasound equipment
- [ ] Ultrasound equipment available but broken
- [ ] Limited training/skills to operate the equipment
- [ ] Insufficient time for scanning during patient consultation
- [ ] Insufficient time for training
- [ ] Inaccessibility of training/Distance from training opportunities
- [ ] Unable to leave clinic for training due to staff shortages/no locum cover
- [ ] Limited time for training due to family/personal commitments
- [ ] Insufficient funding/budget
- [ ] Legal/insurance concerns
- [ ] Limited employer/management support
- [ ] Other (please specify) or any additional comments

University of South Australia

UniSA HEALTHY NEWBORN PROJECT National antenatal ultrasound needs analysis survey

AVAILABILITY OF ANTENATAL ULTRASOUND

*** 14. Why is ultrasound not used when antenatal care is delivered? (select all applicable options)**

- [ ] Ultrasound not clinically indicated/necessary
- [ ] No ultrasound equipment available
- [ ] Difficulty accessing ultrasound equipment
- [ ] Ultrasound equipment available but broken
- [ ] Insufficient time for scanning during patient consultation
- [ ] Equipment available but no trained staff to operate it
- [ ] Insufficient time for training
- [ ] Inaccessibility of training/Distance from training opportunities
- [ ] Unable to leave clinic for training due to staff shortages/no locum cover
- [ ] Limited time for training due to family/personal commitments
- [ ] Insufficient funding/budget
- [ ] Legal/insurance concerns
- [ ] Limited employer/management support
- [ ] Other (please specify) or any additional comments

*** 15. What do you foresee would be the clinical indications for your clinic should Ultrasound become available? (select one option for each indication/row listed below)**

	Common	Uncommon	Not Applicable
Estimated due date	◯	◯	◯
Fetal growth	◯	◯	◯
Identify multiple pregnancy	◯	◯	◯
Placenta location	◯	◯	◯
Positioning- Fetal lie/Presentation	◯	◯	◯
Loss of fetal movement	◯	◯	◯
Identification of fetal heartbeat	◯	◯	◯
Amniotic fluid assessment	◯	◯	◯
Fetal anomalies/defects	◯	◯	◯
History of PV bleeding	◯	◯	◯
Ectopic pregnancy	◯	◯	◯
Maternal trauma/injury/illness	◯	◯	◯

Any additional comments (optional)

University of South Australia

UniSA HEALTHY NEWBORN PROJECT National antenatal ultrasound needs analysis survey

ULTRASOUND EQUIPMENT

*** 16. How many ultrasound machines are available to your clinic?**

- ◯ 0
- ◯ 1
- ◯ 2
- ◯ 3 or more

Any additional comments (optional)

University of South Australia

UniSA HEALTHY NEWBORN PROJECT National antenatal ultrasound needs analysis survey

ULTRASOUND EQUIPMENT

Complete the following questions **regarding your most used piece of equipment**

*** 17. Where is the ultrasound equipment usually located?**

- ◯ Onsite- within the clinic
- ◯ Nearby department/clinic/hospital (loaned for scanning)
- ◯ Brought in by visiting health professional
- ◯ Unknown
- ◯ Other (please specify)

*** 18. Details of equipment**

- ◯ Large ultrasound unit
- ◯ Small portable ultrasound unit

*** 19. Available ultrasound probes (select all applicable options)**

- ☐ Standard transabdominal (TA) probe
- ☐ High resolution transabdominal (TA) probe
- ☐ Transvaginal (TV) probe
- ☐ Unknown

*** 20. Make and model (if known)?**

E.g. Make: Sonosite Model: Edge

- ◯ Unknown
- ◯ Make and model

*** 21. What is the estimated age of your ultrasound machine?**

- ◯ 0-5 years
- ◯ 5-10 years
- ◯ Older than 10 years
- ◯ Unknown

Any additional comments (optional)

*** 22. Is the equipment under a service contract?**

- ◯ Unknown
- ◯ No
- ◯ Yes

*** 23. Has the equipment ever broken down?**

- ◯ Unknown
- ◯ No
- ◯ Yes

If yes, how long did it take to be repaired?

ACCESSIBILITY OF ANTENATAL ULTRASOUND

*** 25. How long do pregnant women in your community have to travel to reach ultrasound services?** **(select one option for each row)**

	1 to 30mins	30mins to 1hr	1 to 3hrs	3 to 6hrs	6 to 12hrs	12 to 24hrs	2 or more days
Shortest travel time	○	○	○	○	○	○	○
Greatest travel time	○	○	○	○	○	○	○
Average travel time	○	○	○	○	○	○	○

Any additional comments (optional)

*** 26. Which method of transportation do pregnant women in your community use to access an ultrasound scan?** **(select all applicable options)**

- ☐ Walk
- ☐ Drive
- ☐ Public transport (Bus/train)
- ☐ Fly

Any additional comments (optional)

*** 27. Is any travel assistance available for pregnant women in your community?**

- ○ Unknown
- ○ No
- ○ Yes (please specify)

*** 28. Is funding available for an accompanying person?**

- ○ Unknown
- ○ No
- ○ Yes (please specify in dollar amount or an estimate of cost)

*** 29. Are there any limitations to the existing travel arrangements?**

- ○ Unknown
- ○ No
- ○ Yes (please specify)

*** 30. Do pregnant women have to pay out of pocket to access ultrasound services?**

- ◯ Unknown
- ◯ No
- ◯ Yes (please specify in dollar amount or an estimate of cost)

*** 31. From your experience, what do you believe some of the barriers may be preventing pregnant women from accessing and utilising antenatal ultrasound services? (select all applicable options)**

- ☐ Ultrasound considered unnecessary
- ☐ Long distances to reach ultrasound service
- ☐ No transport available
- ☐ Cost of travel
- ☐ Cost of the scan
- ☐ No child care options for children at home
- ☐ Unable to get time off work
- ☐ Lack of family support
- ☐ Appointment availability/Convenience of appointment times
- ☐ Other (please specify) or any additional comments

University of South Australia

UniSA HEALTHY NEWBORN PROJECT National antenatal ultrasound needs analysis survey

PERCEPTIONS AND ATTITUDES OF THE HEALTHCARE PRACTITIONER

Please complete the following questions even if your clinic does not offer ultrasound services

*** 32. Antenatal ultrasound is essential to prenatal care?**

- ◯ Strongly agree
- ◯ Agree
- ◯ Neutral
- ◯ Disagree
- ◯ Strongly disagree

Please explain your response

*** 33. In your opinion, why do pregnant women attend for antenatal ultrasound scans? (either point-of-care ultrasound at your clinic or a referred full diagnostic antenatal ultrasound)**

* 34. In your opinion, does having an antenatal ultrasound scan make a difference in pregnant women's lifestyle choices? (i.e. healthier eating, exercise, reduce or stop smoking/drinking etc...)

Yes

If yes, how? [ ]

No

If no, why? [ ]

* 35. What estimated percentage of women attending your clinic request an antenatal ultrasound scan?

- ◯ Offered to all women, no need to request
- ◯ Unknown
- ◯ Percentage requesting scan: [ ]

* 36. Is non-attendance of antenatal appointments a problem in your clinic?

- ◯ No
- ◯ Yes - Please enter estimated average number of missed appointments per month [ ]

* 37. Do pregnant women who attend their appointment refuse to have an ultrasound scan? (either point-of-care ultrasound at your clinic or a referred full diagnostic antenatal ultrasound)

- ◯ No
- ◯ Yes - Please specify estimated number of refusals per year and the reason/s (if given) for declining the scan [ ]

University of South Australia

UniSA HEALTHY NEWBORN PROJECT National antenatal ultrasound needs analysis survey

TRAINING

* 38. Would you be interested in undertaking training in basic antenatal ultrasound scanning? (please indicate your interest regardless of your ability to do so)

- ◯ Yes
- ◯ No

Any additional comments (optional) [ ]

University of South Australia

UniSA HEALTHY NEWBORN PROJECT National antenatal ultrasound needs analysis survey

TRAINING

*Point of Care Ultrasound (POCUS): the use of ultrasound with point of care equipment for diagnostic purposes

*** 39. What ultrasound training and support would benefit your clinic?**

- ☐ Staff sent for off-site POCUS training
- ☐ Onsite (within your clinic) POCUS training
- ☐ Online training
- ☐ Ongoing online mentoring support
- ☐ Onsite supervision and mentoring
- ☐ Refresher courses
- ☐ Full POCUS training and certification (ASUM courses- CAHPU, CCPU)
- ☐ Other (please specify)

*** 40. What would make it easier for you to learn/increase your ultrasound skills?**

*** 41. Would you be willing to travel out of your community/state for antenatal ultrasound training purposes?**

- ◯ Yes
- ◯ No

Any additional comments (optional)

*** 42. What obstacles make it difficult for you to access training? (select all applicable options)**

- ☐ None- There are no significant obstacles
- ☐ Difficulty accessing ultrasound equipment
- ☐ Cost of travel/training
- ☐ Insufficient time to attend training
- ☐ Unable to leave clinic due to staff shortages/no locum cover
- ☐ Inaccessibility of training/Distance from training opportunities
- ☐ Limited time for training due to Family/Personal commitments
- ☐ Limited Employer/Management support
- ☐ Other (please provide detail on barriers not listed and/or any additional comments)

University of South Australia

UniSA HEALTHY NEWBORN PROJECT National antenatal ultrasound needs analysis survey

TRAINING

*** 43. Why are you not interested in undertaking antenatal ultrasound training? (select all applicable options)**

- ☐ Already possess basic skill set
- ☐ Ultrasound is an unnecessary skill for me
- ☐ Insufficient antenatal patients to justify training/maintaining the skill
- ☐ Difficulty accessing ultrasound equipment
- ☐ Cost of travel/training
- ☐ Insufficient time to attend training
- ☐ Insufficient time for scanning during patient consultation
- ☐ Unable to leave clinic for training due to staff shortages/no locum cover
- ☐ Inaccessibility of training/Distance from training opportunities
- ☐ Limited time for training due to Family/Personal commitments
- ☐ Limited Employer/Management support
- ☐ Legal/insurance concerns
- ☐ I would undertake training if offered onsite
- ☐ Other (please provide detail on barriers not listed and/or any additional comments)

University of South Australia

UniSA HEALTHY NEWBORN PROJECT National antenatal ultrasound needs analysis survey

TELEHEALTH

*Telehealth: the remote delivery of health services via video and telecommunications technologies, providing the means for clinicians to communicate face-to-face from different locations including reporting of medical imaging.

*** 44. Is Telehealth available in your clinic?**

- ○ No
- ○ Yes

Please specify what Telehealth is currently being used for in your clinic:

*** 45. Can you see a potential for using Telehealth with ultrasound?**

Yes

Please specify why:

No

Please specify why:

University of South Australia

UniSA HEALTHY NEWBORN PROJECT National antenatal ultrasound needs analysis survey

FINAL COMMENT

46. Do you have any additional comments in regards to the provision of ultrasound services to pregnant women in rural/remote Australia?

University of South Australia

UniSA HEALTHY NEWBORN PROJECT National antenatal ultrasound needs analysis survey

Thank you for completing the survey

To enter the draw to win the **$1000 scholarship prize**, please click the following link to be redirected to the University of South Australia's website to securely provide your contact details. These detail are not connected to your survey responses.

https://www.unisa.edu.au/Health-Sciences/Schools/Health-Sciences/Research/Healthy-Newborn-Project/
